# Continuous Glucose Monitoring–Derived Metrics and Cardiovascular Risk Among People With Diabetes: Systematic Scoping Review

**DOI:** 10.2196/89374

**Published:** 2026-05-06

**Authors:** Helene Bei Thomsen, Benjamin Lebiecka-Johansen, Ole Nørgaard, Tue Helms Andersen, Signe Toft Andersen, Guy Fagherazzi, Adam Hulman, Anders Aasted Isaksen

**Affiliations:** 1 Steno Diabetes Center Aarhus, Palle Juul-Jensens Boulevard 11, Aarhus, Central Denmark Region, 8200, Denmark, +45 23 70 74 81; 2 Department of Public Health, Aarhus University, Aarhus, Central Denmark Region, Denmark; 3 Danish Diabetes Knowledge Center, Department of Education, Copenhagen University Hospital - Steno Diabetes Center Copenhagen, Herlev, Denmark; 4 Medical Department, Gødstrup Hospital, Herning, Central Denmark Region, Denmark; 5 Department of Precision Health, Deep Digital Phenotyping Research Unit, Luxembourg Institute of Health, Strassen, Luxembourg

**Keywords:** diabetes, blood glucose, cardiovascular disease, cardiovascular risk, continuous glucose monitoring, scoping review, prediction, association

## Abstract

**Background:**

Conventional clinical markers guide cardiovascular risk stratification; however, continuous glucose monitoring (CGM) data remain absent from prediction models. A synthesis of the current literature is needed to clarify the prognostic relevance of CGM data for cardiovascular outcomes in people with diabetes.

**Objective:**

This scoping review aimed to identify published studies examining (1) the associations between glycemic control and cardiovascular outcomes and (2) the predictive value of CGM-derived metrics in cardiovascular risk assessment.

**Methods:**

MEDLINE and Embase were searched from inception to March 11, 2025, for peer-reviewed, original research that included CGM-derived metrics and cardiovascular disease (CVD) outcomes. Two reviewers screened the records independently.

**Results:**

A total of 53 studies were identified. These studies focused on type 1 diabetes, type 2 diabetes, both diabetes types, or prediabetes. Clinical outcomes were examined in 16 studies, while subclinical outcomes were assessed in 40 studies. Of the 53 studies, 47 were cross-sectional studies and 6 were longitudinal studies. All studies were association studies, and 3 included secondary analyses of predictive performance. However, none applied machine learning–based methods. A wide range of CGM-derived metrics and CVD outcomes, both clinical and subclinical, were studied in the literature.

**Conclusions:**

Overall, the findings were inconsistent across studies, and this was likely due to methodological weaknesses such as underpowered analyses. Time-in-range was both the most studied metric and associated with cardiovascular risk in the largest single study. Only the mean amplitude of glycemic excursions was consistently associated with CVD in most studies investigating this metric, when using statistical significance as a pragmatic indicator of consistency across heterogeneous studies. The prognostic value of CGM-derived metrics for CVD outcomes is currently underexplored. Longitudinal prediction studies on clinical CVD outcomes, leveraging the potential of routinely collected CGM data, are needed.

## Introduction

Cardiovascular disease (CVD) is the main cause of disability and mortality among people with diabetes [1]. Abundant literature exists on the use of simple clinical measurements for risk prediction models to identify individuals at high risk of developing CVD [2,3]. However, these prediction models are rarely used in clinical practice due to methodological flaws and a lack of external validation [4]. However, exceptions do exist, but they are limited to the use of traditional risk factors such as sex, age, smoking status, and routinely collected biomarkers [5,6].

Progress has been made in developing digital tools and wearable technologies, such as continuous glucose monitoring (CGM) devices, to aid decisions in diabetes management [7,8]. CGM has been shown to be an effective tool for achieving glycemic control [9]. The barriers to CGM usage have mostly been overcome [10], and it is expected that the use of CGM devices will rise as sensors become less obtrusive and more cost-effective [10,11]. This will lead to the accumulation of a large amount of CGM data that may hold predictive potential for CVD prediction, given advances in artificial intelligence and the established links between glycemic control measured by hemoglobin A_1c_ (HbA_1c_) and CVD risk [12,13]. The predictive aspect has been overlooked in previous efforts to synthesize evidence on the links between CGM data, including CGM-derived metrics, and CVD complications [14,15]. There should be a focus on the distinction between association and prediction, since biomarkers with strong associations can exhibit modest predictive value for risk stratification in precision medicine [16].

Therefore, the objective of this scoping review was to identify studies focusing on CGM-derived metrics as predictors of CVD and assess associations between glycemic control and CVD risk in people with diabetes.

## Methods

### Scoping Review Framework and Reporting

This scoping review has been conducted according to the Manual for Evidence Synthesis (Chapter 10 - Scoping reviews) from the Joanna Briggs Institute [17] and reported according to the PRISMA-ScR (Preferred Reporting Items for Systematic Reviews and Meta-Analyses extension for Scoping Reviews) guidelines (Checklist 1) [18]. A detailed study protocol for this scoping review has previously been published, along with a description of any deviations from the original protocol [19].

### Concepts and Definitions

In this review, the term “CGM-derived metrics” covers all metrics derived from CGM device data. Blood glucose metrics like HbA_1c_ or measurements from anything other than CGM data will not be included (eg, metrics based on self-monitored blood glucose measurements taken with finger-prick or measurements from blood samples such as HbA_1c_). CVD outcomes were grouped as either clinical or subclinical. The following outcomes were considered clinical CVD: cardiovascular mortality, major adverse cardiovascular events, coronary artery disease, heart failure, stroke, and peripheral artery disease. Synonymous terms (eg, ischemic heart disease) and clinical events (eg, undergoing coronary artery bypass surgery) were also included.

Subclinical outcome measures were grouped into 6 subcategories: arterial stiffness, flow resistance, arterial wall thickness, arterial wall composition, cardiac and pulse-related measures, and arterial lumen. CVD outcomes did not include broader risk factors nonspecific to cardiovascular risk (eg, age and sex).

### Eligibility Criteria

The eligibility criteria are reported in Textbox 1, with further details provided in the review protocol [19].

Textbox 1. **Eligibility criteria.**

**Inclusion criteria**

- Human clinical studies including participants with prediabetes or any type of diabetes, except for gestational diabetes, regardless of definitions.
- Peer-reviewed published original articles (including brief reports).
- Studies investigating either:
  - The association between continuous glucose monitoring (CGM)-derived metrics of glycemic control and cardiovascular risk markers or cardiovascular diseases (prevalent or incident).
  - CGM-derived metrics of glycemic control as predictors of cardiovascular risk markers or cardiovascular diseases (prevalent or incident).

**Exclusion criteria**

- Review articles, editorials, case reports, protocols, conference abstracts, and preprints.
- Animal studies not including any human participants.
- Studies not including metrics derived from CGM device data.
- Studies including CGM-derived metrics as outcomes.
- Studies not focusing on cardiovascular disease outcomes according to our definition, as outlined in the Concepts and Definitions section.
- Studies focusing on pregnant women with any form of diabetes, including gestational diabetes.
- Studies where participants were monitored after surgery or during hospitalization (eg, intensive care unit).
- Language not understood by the authors.

### Information Sources and Search

The MEDLINE and Embase databases were searched from inception to March 11, 2025, using a search strategy tested against 13 key articles within the field [12,14,20-30] by an information specialist (ON) and reviewed by another (THA; Multimedia Appendix 1) [31].

### Selection of Sources of Evidence

Following the search, all identified citations were collated and uploaded into EPPI Reviewer 6, and duplicates were removed [32]. A meeting was held after about 5% of all titles and abstracts had been screened to create consistency among the reviewers (HBT, BL-J, AH, and AAI). In the screening phase, 2 independent reviewers screened the titles and abstracts to assess eligibility. When all titles and abstracts had been screened, full-text versions of relevant articles were retrieved and assessed in detail against the eligibility criteria by 2 independent reviewers. The reasons for exclusion during full-text screening were recorded and reported. Any disagreements that arose between the reviewers at any stage of the selection process were resolved through discussion. Disagreements unresolved through discussion were settled by the senior researcher (AH).

After the first screening phase, the software tool citationchaser was used for backward and forward citation searching [31]. The tool was applied to all included studies, and the screening process was repeated until no additional studies were found through backward citation and forward citation searching [19].

### Data Charting Process and Data Items

Research questions were predefined and published in the scoping review protocol (Textbox 2) [19], and a corresponding data extraction table was developed based on the PRISMA-ScR checklist [18]. Data were extracted by the first author (HBT) and verified by the last author (AAI).

Textbox 2. **Research questions.**

1. Is there an association between glycemic control and cardiovascular disease (CVD) risk?
2. Can continuous glucose monitoring (CGM)-derived metrics predict CVD risk?
3. What CGM-derived metrics are used in the literature?
4. Which cardiovascular markers and diseases are included as outcomes in the studies?
5. What characterizes study populations (age, sex, ethnicity, or geographic location)?
6. What study designs are used (eg, longitudinal cohort, randomized controlled trial, and cross-sectional)?
7. How was data collected (eg, clinical trial, epidemiological study, and routinely collected data)?
8. What CGM devices were used?
9. What statistical models were used in the studies?
10. Are the data openly available?
11. Is the code openly available?

### Synthesis of Results

Study characteristics were aggregated using descriptive statistics, and a narrative summary accompanied the tabulated results.

We considered the most adjusted models to be more clinically relevant and therefore extracted results only from these in the main population of each study, if studies reported numerous estimates due to multiple adjustment levels, subgroup stratifications, and varied combinations of CGM metrics and CVD outcomes. Results are reported for all studies, both clinical and subclinical, but only the specific details of association studies investigating clinical outcomes have been prioritized and presented in the main text, and evidence from the subclinical studies has been presented in Multimedia Appendix 2. A full list of all the CGM metrics and CVD outcomes found in the literature is presented in Multimedia Appendix 3.

## Results

### Study Selection Process

**Figure 1.**
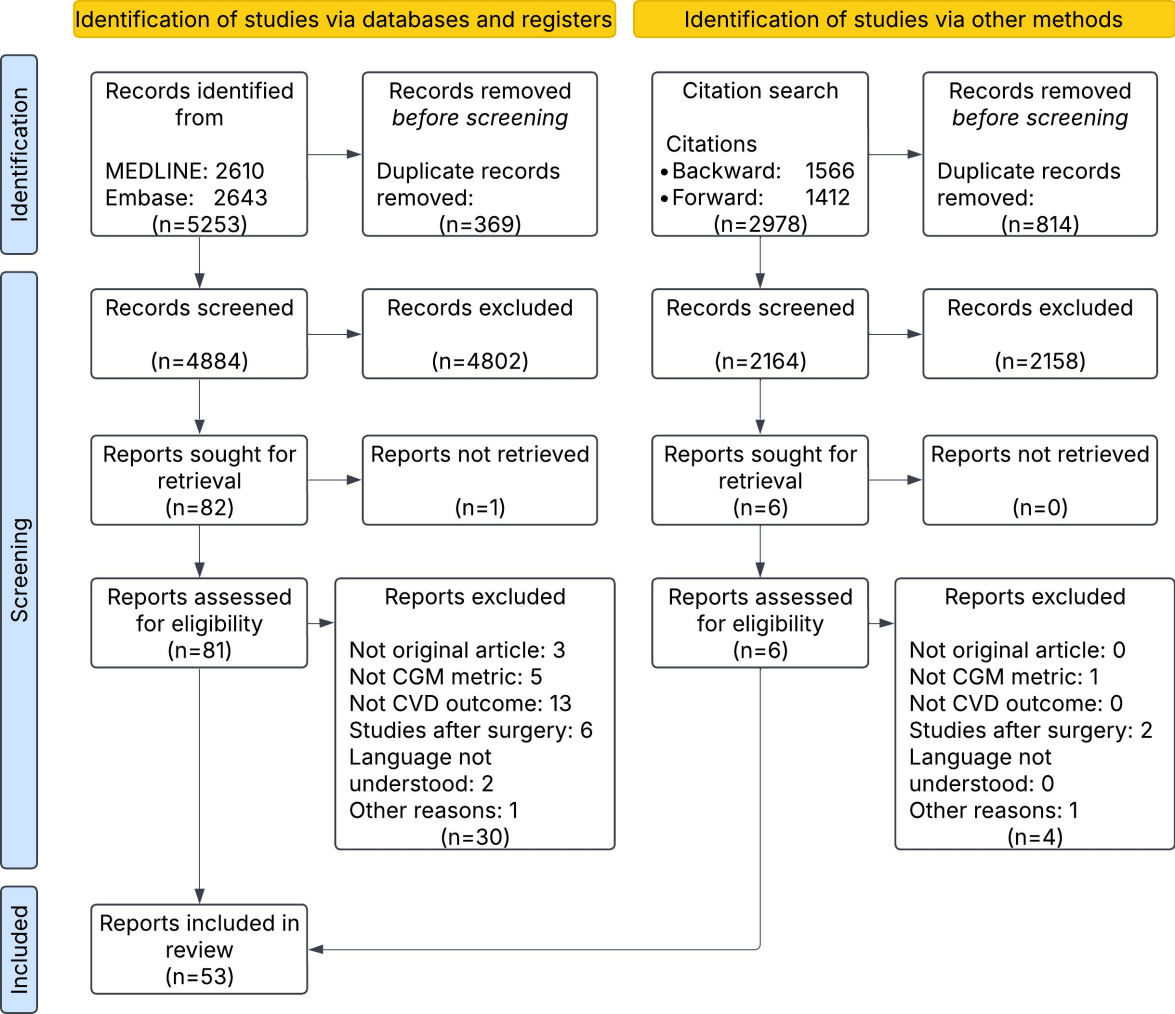
Flow diagram of study selection. CGM: continuous glucose monitoring; CVD: cardiovascular disease.

The search identified 5253 records, of which 369 were duplicates and therefore removed (Figure 1). After title and abstract screening, 4802 records were excluded, leaving 82 records for full-text screening.

For full-text screening, 1 study could not be retrieved and 30 studies were excluded for the following reasons: not an original article (n=3), CGM-derived metrics were not based on CGM data (n=5), the patient group did not have CVD as an outcome (n=13), the study was in the postsurgery stage (n=6), the study language was not understood by the authors of this review (n=2), and the study was not yet published in a journal (n=1). Two additional studies were identified through backward and forward citation searching, resulting in a total of 53 included studies on clinical and subclinical outcomes (MultimediaAppendices 4  5).

### Study Populations

The most common patient group was people with type 2 diabetes (34 out of 53 studies), followed by people with type 1 diabetes (20 out of 53 studies) and those with prediabetes (4 out of 53 studies). The patient group mostly included adults, with 7 studies focusing on children younger than 18 years [33-39]. Almost all studies included both male and female participants, with the exception of 2 studies [40,41], which included only male participants. The geographic distribution of the studies was uneven, as most studies were from Asia (27 out of 53 studies) and Europe (22 out of 53 studies). Three studies included data collected in North America, and 1 study was from Australia. Only 1 study included data from an African country; however, the study population was still predominantly White [42]. No studies were from South America (Multimedia Appendix 6).

### Study Designs and Data Collection

One study was a randomized controlled trial [34], and the remaining 52 were observational studies. The majority of studies (49 out of 53 studies) performed cross-sectional analyses, and only 5 studies conducted longitudinal analyses. The size of the study population varied greatly, ranging from 17 to 6225, with a median of 152 (IQR 75‐469). Eleven studies included routinely collected CGM data from the participants’ own devices, and 42 studies used CGM data that had been actively collected with a device provided as part of the study. Studies analyzed data with Spearman or Pearson correlation (n=11), Cox proportional hazards regression (n=3), linear regression (n=23), or logistic regression (n=22; MultimediaAppendices 7  8). All studies in this review were association studies, with only 3 studies reporting prediction measures from secondary analyses [28,43,44], and none of these 3 studies used machine learning methods. None of the studies shared data or code.

### CGM Findings

Medtronic devices were most frequently used (28 out of 53 studies), followed by Abbott (9 out of 53 studies) and Dexcom (5 out of 53 studies). Other companies were Menarini, Meiqi Company, and SIBIONICS (Multimedia Appendix 7). Sampling frequencies varied from 3 to 15 minutes but were only reported in a minority of studies (21 out of 53 studies).

Among the 53 studies, the most common CGM-derived metrics were time in range (TIR; n=23), mean amplitude of glycemic excursions (MAGE; n=22), mean blood glucose (n=21), SD (n=19), coefficient of variation (CV; n=19), time below range (TBR; n=16), and time above range (TAR; n=15; Multimedia Appendix 9). Most studies did not find a statistically significant association between CGM-derived metrics and CVD. For example, only 7 out of 23 (30%) studies involving TIR found an association between TIR and CVD. However, among studies involving MAGE, a high proportion (14/22, 64%) reported an association between MAGE and CVD. The use of CGM-derived metrics differed among studies involving different diabetes populations. For example, 10 out of 23 (43%) studies involving TIR and 5 out of 22 (23%) studies involving MAGE had populations with type 1 diabetes. Among studies on people with type 1 diabetes, limited studies detected a statistically significant association between CGM-derived metrics and CVD (eg, TIR: 0/10, 0%; MAGE: 2/5, 40%). In contrast, studies on people with type 2 diabetes more often detected a statistically significant association (eg, TIR: 5/11, 45%; MAGE: 10/14, 71%). Studies on people with type 2 diabetes had a larger median sample size (TIR: 510, IQR 405-600; MAGE: 251, IQR 89-411) than studies on people with type 1 diabetes (TIR: 214, IQR 119-547; MAGE: 57, IQR 30-215; Multimedia Appendix 9).

### Prediction Studies

Three studies included predictive analyses investigating MAGE as a predictor of CVD using logistic regression models. The reported area under the receiver operating characteristic curve (AUC) was 0.61 in one study [43] and 0.62 in another study [28]. Both studies reported MAGE to be a superior predictor when compared to HbA_1c_, which had AUC values of 0.55 and 0.58, respectively. In the third study, a receiver operator characteristic curve analysis was conducted to ascertain the optimal threshold for dichotomizing MAGE as part of the variable selection process, but the AUC was not reported [44]. The authors found that MAGE ≥3.4 mmol/L was a risk factor for stenosis and/or occlusion, with a sensitivity of 0.60 and a specificity of 0.61.

### Association Studies

Of the 53 included studies, 13 (25%) focused solely on clinical outcomes, 37 (70%) focused solely on subclinical outcomes, and 3 (6%) investigated both outcomes. The design/demographics and main findings of the studies on clinical cardiovascular outcomes are summarized in Tables1  2, respectively, and the results of the studies on subclinical cardiovascular outcomes are summarized in Multimedia Appendix 7.

**Table 1.** Study design and demographics of the included studies on clinical cardiovascular outcomes.

Reference	Study design	Population	Size, n	Age^a^ (years)	Diabetes duration^b^ (years)	HbA_1c_^c^ (mmol/mol, %)	CGM^d^ duration
Chen et al [40]^e^, 2020	Longitudinal (prospective) and cross-sectional (retrospective); FU^f^: in-hospital or within 3 months after discharge from hospital	T2D^g^ with CAD^h^ (only male); BG^i^ control: n=90, BG fluctuation: n=120	210	BG control: mean 55.53 (SD 7.30), BG fluctuation: mean 56.41 (SD 7.67)	BG control: mean 6.59 (SD 2.30), BG fluctuation: mean 6.92 (SD 2.25)	—^j^	2 days
He et al [45], 2023	Longitudinal (prospective); FU: 1 year	T2D with kidney disease on hemodialysis; High TIR^k^: n=12, Low TIR: n=15	27	High TIR: median 66 (IQR 63-73), Low TIR: median 70 (IQR 64-75)	High TIR: median 2 (IQR 1.7-10), Low TIR: median 5 (IQR 0.75-20)	High TIR: median 43 (IQR 37-51) mmol/mol or 6.1% (IQR 5.5%-6.8%), Low TIR: median 66 (IQR 46-70) mmol/mol or 8.2% (IQR 6.4%-8.6%)	14 days
Lu et al [21], 2021	Longitudinal (prospective); FU: until death occurred or 3‐13 years, median: 6.9 years	T2D; Hospitalized	6225	Mean 61.7	Mean 9.7	Mean 74.0 (SD 24.0) mmol/mol or mean 8.9% (SD 2.2%)	72 hours
Wei et al [46], 2019	Longitudinal (prospective); Median FU: 31 (IQR 22-56) months	T2D; Divided into three groups: (1) No hypoglycemia, n=1173; (2) Mild hypoglycemia (level 1), n=323; (3) Severe hypoglycemia (level 3), n=24	1520	No hypoglycemia: mean 58.59 (SD 11.26), Hypoglycemia: mean 62.27 (SD 11.58)	No hypoglycemia: mean 6.46 (SD 6.00), Hypoglycemia: mean 7.78 (SD 7.37)	No hypoglycemia: mean 8.19% (SD 2.10%), Hypoglycemia: mean 7.73% (SD 1.96%)	3 days
Bezerra et al [47], 2023	Cross-sectional	T1D^l^	161	Mean 37.4 (SD 13.4)	Mean 17.7 (SD 10.6)	Mean 7.5% (SD 1.1%)	14 days
De Meulemeester et al [48]^e^, 2024	Cross-sectional	T1D	808	Mean 44.8 (SD 15.2)	Mean 23.1 (SD 13.6)	Mean 63 (SD 13) mmol/mol or 7.9% (SD 1.2%)	2 weeks
Deng et al [49], 2023	Cross-sectional	T2D	860	Hp1 carriers: mean 53.5 (SD 13.3), Hp2‐2: mean 51.7 (SD 14.6)	Hp1 carriers: mean 8.6 (SD 6.4), Hp2‐2: mean 8.6 (SD 6.7)	Hp1 carriers: mean 73.0 (SD 24.0) mmol/mol or 8.8% (SD 2.2%), Hp2-2: mean 72.0 (SD 23.0) mmol/mol or 8.7% (SD 2.1%)	3 days
El Malahi et al [50], 2022	Cross-sectional	T1D starting on sensor-augmented pump therapy	515	Mean 42.2 (SD 12.5)	Mean 22.3 (SD 11.6)	Mean 60 (SD 9.8) mmol/mol or 7.6% (SD 0.9%)	2 weeks
Guo et al [51], 2021	Cross-sectional	T1D or T2D with atrial fibrillation; With stroke: n=48, Without stroke: n=462	510	Stroke: mean 70.3 (SD 12.1), No stroke: mean 68.1 (SD 9.4)	—	Stroke: mean 8.2 (SD 1.7), No stroke: mean 7.4 (SD 2.1)	72 hours
Li et al [52], 2020	Cross-sectional	T2D with LEAD^m^: n=179, T2D without LEAD: n=157	336	With LEAD: mean 65.56 (SD 11.99), Without LEAD: mean 55.94 (SD 12.45)	With LEAD: mean 10.32 (SD 4.14), Without LEAD: mean 6.92 (SD 3.54)	With LEAD: mean 8.97% (SD 1.63%), Without LEAD: mean 7.85% (SD 1.41%)	72 hours
Magri et al [27]^e^, 2018	Cross-sectional	T2D	121	Median 64 (IQR 57-68)	Median 3 (IQR 2-5)	Median 45 mmol/mol (6.8%)	72 hours
Shu-Hua et al [43], 2012	Cross-sectional	T2D with chest pain; Without CAD: n=202, With CAD, n=84	286	Without CAD: mean 62.8 (SD 8.7), With CAD: mean 66.6 (SD 9.2)	—	Without CAD: mean 7.51% (SD 0.80%), With CAD: mean 7.75% (SD 0.92%)	72 hours; Only used the intermediate 48 hours
Sheng et al [53], 2023	Cross-sectional	T2D; Hospitalized	545	Mean 61.22 (SD 11.21)	—	Mean 8.51% (SD 1.85%)	7‐14 days
Su et al [28], 2011	Cross-sectional	T2D with chest pain; Without CAD: n=92, With CAD: n=252	344	Without CAD: mean 61 (SD 9), With CAD: mean 65 (SD 9)	Without CAD: mean 4.8 (SD 5.7), With CAD: mean 6.5 (SD 6.4)	Without CAD: mean 7.5% (SD 1.4%), With CAD: mean 7.6% (SD 1.5%)	72 hours; Only 48 hours used
Watanabe et al [54], 2017	Cross-sectional	Prediabetes; Hospitalized	28	Mean 64.3 (SD 12.8)	—	Mean 5.41% (SD 0.35%)	72 hours; Only used the middle 48 hours
Zhang et al [30], 2013	Cross-sectional	T2D with cardiovascular complications; Group A: healthy individuals, Group B: T2D without cardiovascular complications, Group C: T2D with cardiovascular complications	92	Group A: mean 56.3 (SD 6.1), Group B: mean 56.1 (SD 6.6), Group C: mean 61.7 (SD 7.2)	—	Group A: mean 5.3% (SD 0.3%), Group B: mean 6.6% (SD 1.2%), Group C: mean 7.5% (SD 1.4%)	72 hours

a Age is reported as an interval, mean (SD), or median (IQR).

b Diabetes duration values originally reported in months were converted to years for consistency (months ÷ 12).

c HbA_1c_: hemoglobin A_1c_.

d CGM: continuous glucose monitoring.

e This study appears in both the clinical and subclinical disease outcome tables owing to the investigation of multiple cardiovascular disease outcomes.

f FU: follow-up.

g T2D: type 2 diabetes.

h CAD: coronary artery disease.

i BG: blood glucose.

j Not available or not reported.

k TIR: time in range.

l T1D: type 1 diabetes.

m LEAD: lower extremity arterial disease.

**Table 2.** Main findings of the included studies on clinical cardiovascular outcomes^a^.

Outcome, reference, and continuous glucose monitoring metrics	Unadjusted or least adjusted findings^b^	*P* value for the least adjusted findings	Most adjusted findings^b^	*P* value for the most adjusted findings
Cardiovascular mortality				
Lu et al [21], 2021				
TIR^c^ >85%	HR 1.00	<.001 (trend)	HR 1.00	.02 (trend)
TIR 71%‐85%	HR 1.43 (0.95‐2.14)	<.001 (trend)	HR 1.35 (0.90-2.04)	.02 (trend)
TIR 51%‐70%	HR 1.66 (1.12‐2.45)	<.001 (trend)	HR 1.47 (0.99-2.19)	.02 (trend)
TIR ≥50%	HR 2.15 (1.47‐3.13)	<.001 (trend)	HR 1.85 (1.25-2.72)	.02 (trend)
TIR as a continuous variable (each 10% decrease)	HR 1.08 (1.03‐1.13)	—^d^	HR 1.05 (1.00‐1.11)	—
Wei et al [46]^e^, 2019				
Hypoglycemic events	HR 2.033 (1.211-3.413)	—	HR 2.642 (1.398-4.994)	—
Major adverse cardiovascular events				
He et al [45], 2023				
Blood glucose risk index	HR 0.97 (0.85-1.10)	.61	HR 0.98 (0.85-1.13)	.75
Low blood glucose index	HR 2.37 (1.16-4.83)	.02	HR 2.73 (1.21-6.16)	.02
High blood glucose index	HR 0.94 (0.81-1.08)	.38	HR 0.94 (0.81-1.09)	.44
Average of daily risk range	HR 1.00 (0.93-1.07)	>.99	HR 1.01 (0.93-1.09)	.80
GMI^f^	HR 0.98 (0.91-1.06)	.65	HR 0.99 (0.91-1.07)	.78
M-value	HR 0.98 (0.91-1.05)	.54	HR 0.98 (0.91-1.06)	.64
Wei et al [46]^e^, 2019				
Hypoglycemic events	HR 1.501 (1.207-1.866)	—	HR 1.615 (1.239-2.106)	<.001
Macrovascular complications				
De Meulemeester et al [48]^e^^,^^g^, 2024				
TIR	OR 0.939 (0.829-1.063)	>.05	OR 0.896 (0.738-1.087)	>.05
TITR^h^	OR 0.901 (0.775-1.047)	>.05	OR 0.933 (0.745-1.169)	>.05
Bezerra et al [47], 2023				
TIR	OR 0.66 (0.46‐0.93)	.02	OR 0.68 (0.39-1.16)	.15
Time below 54 mg/dL	OR 1.10 (0.88‐1.38)	.39	OR 0.92 (0.62-1.34)	.65
TBR^i^	OR 0.93 (0.80‐1.09)	.39	OR 0.77 (0.54-1.11)	.17
TAR^j^	OR 1.04 (1.01‐1.08)	.01	OR 1.04 (0.99-1.10)	.08
Time above 250 mg/dL	OR 1.04 (1.00‐1.08)	.03	OR 1.03 (0.97-1.09)	.29
CV^k^	OR 1.03 (0.95‐1.11)	.52	OR 0.92 (0.81-1.06)	.25
GMI	OR 2.17 (1.14‐4.11)	.02	OR 2.03 (0.77-5.37)	.15
Deng et al [49], 2023				
%CV tertile 1 (Hp1; reference)	OR 1.000	.07 (interaction)	OR 1.000	.008 (interaction)
%CV tertile 1 (Hp2‐2; reference)	OR 1.000	.07 (interaction)	OR 1.000	.008 (interaction)
%CV tertile 2 (Hp1)	OR 1.483 (0.907-2.423)	.12	OR 1.048 (0.528-2.078)	.89
%CV tertile 2 (Hp2‐2)	OR 1.399 (0.829-2.358)	.21	OR 0.659 (0.296-1.466)	.31
%CV tertile 3 (Hp1)	OR 2.347 (1.393-3.957)	.001	OR 2.461 (1.183-5.121)	.02
%CV tertile 3 (Hp2‐2)	OR 1.217 (0.731-2.027)	.45	OR 0.540 (0.245-1.191)	.13
El Malahi et al [50], 2022				
TIR	—	—	—	>.05
SD	—	—	—	>.05
CV	—	—	—	>.05
Magri et al [27]^g^, 2018				
TBR	—	—	OR 1.12 (1.014‐1.228)	.02
Lowest BG^l^ value	—	—	—	—
Area under the TBR curve	—	—	—	—
Coronary artery disease				
De Meulemeester et al [48]^e^^,^^g^, 2024				
TITR	OR 1.039 (0.812-1.330)	>.05	OR 1.255 (0.874-1.803)	>.05
TIR	OR 1.072 (0.866-1.328)	>.05	OR 1.164 (0.844-1.607)	>.05
Sheng et al [53], 2023				
TIR <20%	—	—	OR 2.143 (1.554‐3.287)	—
TIR 20‐40%	—	—	OR 1.049 (0.945‐2.022)	—
TIR 40‐60%	—	—	OR 0.854 (0.495‐1.473)	—
TIR 60‐80%	—	—	OR 0.617 (0.423‐1.312)	—
TIR >80%	—	—	OR 0.470 (0.143‐1.545)	—
Chen et al [40]^e^^,^^g^, 2020				
Controls with SD <1.40 mmol/L, MAGE^m^ <3.90 mmol/L, LAGE^n^ <4.40 mmol/L, MODD^o^ <0.83 mmol/L versus high BG fluctuations (myocardial Infarction)	—	—	𝜒^2^=5.797	.02
Controls with SD <1.40 mmol/L, MAGE <3.90 mmol/L, LAGE <4.40 mmol/L, MODD <0.83 mmol/L versus high BG fluctuations (angina pectoris)	—	—	𝜒^2^=7.490	.006
Wei et al [46]^e^, 2019				
Hypoglycemic events (myocardial Infarction)	HR 1.901 (1.067-3.389)	—	HR 1.549 (0.768-3.124)	.03
Hypoglycemic events (unstable angina pectoris)	HR 1.226 (0.857-1.753)	—	HR 1.218 (0.794-1.869)	.30
Shu-Hua et al [43]^e^, 2012				
MAGE level (≥3.4 mmol/L)	—	—	OR 2.286 (1.176-4.446)	.02
Su et al [28]^e^, 2011				
MAGE ≥3.4 mmol/L	—	—	OR 2.612 (1.423-4.831)	.002
MAGE	—	—	AUC 0.618 (0.555-0.680)	.001
Gensini score				
Chen et al [40]^e^^,^^g^, 2020				
Controls with SD <1.40 mmol/L, MAGE <3.90 mmol/L, LAGE <4.40 mmol/L, MODD <0.83 mmol/L versus high BG fluctuations	—	—	𝘵=6.210	<.001
Watanabe et al [54]^e^, 2017				
MAGE	—	—	*r*=0.742	<.001
Shu-Hua et al [43]^e^, 2012				
MAGE	—	—	Unstandardized coefficient β=4.817; SE=1.614; standardized coefficient β=0.170; *t*=2.984	.003
Su et al [28]^e^, 2011				
MAGE	—	—	Unstandardized β=7.010; SE=1.466; standardized β=0.237; *t*=4.783	<.001
Syntax score				
Watanabe et al [54]^e^, 2017				
MAGE	—	—	*r*=0.776	<.001
Zhang et al [30]^e^, 2013				
MAGE	—	—	*r*=0.518	.01
BG fluctuations from 00:00 to 03:00	—	—	*r*=−0.442	.04
BG fluctuations from 03:00 to 06:00	—	—	*r*=−0.208	.34
BG fluctuations from 06:00 to 08:00	—	—	*r*=0.678	<.001
BG fluctuations from 08:00 to 11:00	—	—	*r*=0.115	.60
BG fluctuations from 11:00 to 13:00	—	—	*r*=0.523	.01
BG fluctuations from 13:00 to 17:00	—	—	*r*=0.257	.24
BG fluctuations from 17:00 to 19:00	—	—	*r*=0.358	.09
BG fluctuations from 19:00 to 24:00	—	—	*r*=−0.018	.93
Stroke				
De Meulemeester et al [48]^e^^,^^g^, 2024				
TITR	OR 0.651 (0.470-0.902)	<.05	OR 0.546 (0.347-0.858)	<.01
TIR	OR 0.749 (0.588-0.955)	<.05	OR 0.617 (0.440-0.866)	<.01
Guo et al [51], 2021				
TIR: Q1 (≤46%; reference)	OR 1.00	<.001	OR 1.00	<.001
TIR: Q2 (46%‐65%)	OR 0.86 (0.72-0.95)	<.001	OR 0.80 (0.68-0.92)	<.001
TIR: Q3 (65%‐81%)	OR 0.71 (0.61-0.81)	<.001	OR 0.64 (0.53-0.79)	<.001
TIR: Q4 (>81%)	OR 0.66 (0.58-0.80)	<.001	OR 0.59 (0.50-0.74)	<.001
TIR (per 10% increase)	OR 0.93 (0.85-0.98)	.008	OR 0.89 (0.82-0.95)	.001
Wei et al [46]^e^, 2019				
Hypoglycemic events	HR 1.691 (1.144-2.499)	—	HR 1.813 (1.110-2.960)	.06
Peripheral artery disease				
De Meulemeester et al [48]^e^^,^^g^, 2024				
TITR	OR 0.680 (0.426-1.085)	>.05	OR 0.807 (0.382-1.703)	>.05
TIR	OR 0.736 (0.520-1.042)	>.05	OR 0.811 (0.470-1.398)	>.05
Lower extremity arterial disease				
Li et al [52], 2020				
TIR	OR 0.979 (0.968-0.991)	<.001	OR 0.979 (0.965-0.992)	.002
CV	OR 1.040 (1.003-1.078)	.04	OR 1.038 (0.996-1.081)	.08
SD	OR 1.325 (1.038-1.691)	.02	OR 1.158 (0.824-1.627)	.40
TIR-without LEAD^p^ (1)	OR 1.00	—	OR 1.00	—
TIR-mild LEAD (1)	OR 0.98 (0.97-1.00)	.14	OR 0.99 (0.97-1.01)	.25
TIR-moderate LEAD (1)	OR 0.97 (0.95-0.99)	.007	OR 0.97 (0.95-0.99)	.01
TIR-without severe LEAD (1)	OR 0.96 (0.94-0.98)	.002	OR 0.96 (0.94-0.98)	.003
CV-without LEAD	—	—	OR 1.00	—
CV-mild LEAD	—	—	OR 1.03 (0.98-1.07)	.28
CV-moderate LEAD	—	—	OR 1.02 (0.96-1.09)	.48
CV-without severe LEAD	—	—	OR 1.02 (0.95-1.09)	.60
TIR-without LEAD (2)	—	—	OR 1.00	—
TIR-mild LEAD (2)	—	—	OR 0.97 (0.96-1.08)	.06
TIR-moderate LEAD (2)	—	—	OR 0.98 (0.95-0.99)	.01
TIR-without severe LEAD (2)	—	—	OR 0.97 (0.95-0.99)	.02
SD-without LEAD	—	—	OR 1.00	—
SD-mild LEAD	—	—	OR 0.88 (0.47-1.64)	.69
SD-moderate LEAD	—	—	OR 1.28 (0.58-3.07)	.58
SD-without severe LEAD	—	—	OR 1.52 (0.92-2.41)	.10

a The full table with adjustments is provided in Multimedia Appendix 10. Further elaboration on the adjusted variables can be found in MultimediaAppendices 7  8.

b All hazard ratios (HRs), odds ratios (ORs), and areas under the curve (AUCs) are reported as follows: point estimate (95% CI).

c TIR: time in range.

d Not applicable or not available/not reported.

e This study appears multiple times as it investigated multiple cardiovascular disease outcomes.

f GMI: glucose management indicator.

g This study appears in both the clinical and subclinical disease outcome tables owing to the investigation of multiple cardiovascular disease outcomes.

h TITR: time in tight range.

i TBR: time below range.

j TAR: time above range.

k CV: coefficient of variation.

l BG: blood glucose.

m MAGE: mean amplitude of glycemic excursions.

n LAGE: largest amplitude of glycemic excursions.

o MODD: mean of daily differences.

p LEAD: lower extremity arterial disease.

### Cardiovascular Mortality

Two longitudinal studies found an association between a CGM-derived metric (TIR and hypoglycemia) and cardiovascular mortality [21,46].

### Major Adverse Cardiovascular Events

Two studies assessed major adverse cardiovascular events [45,46]. Both studies included nonfatal myocardial infarction, nonfatal stroke, and cardiovascular death. One study also included unstable angina leading to hospitalization [46]. Hypoglycemic events and low blood glucose index values were associated with major adverse cardiovascular events; however, no associations were found for other CGM-derived metrics, including glucose management indicator, high blood glucose index, average of daily risk range, m-value, and blood glucose risk index.

### Macrovascular Complications

Five studies explored the association between CGM-derived metrics and nonfatal cardiovascular events regardless of anatomical location as a composite CVD outcome, with some variation between the studies in terms of the complications included [27,47-50]. All 5 studies included cerebrovascular accident, 4 included peripheral artery disease [47-50], 3 included coronary artery disease [48-50], 2 included ischemic heart disease [27,47], and 1 each included stenosis [48], heart failure [50], and ankle-brachial index <0.9 or abnormal carotid intima-media thickness [27]. One study [27] found an association between TBR and cardiovascular complications, while another study [47] did not find an association. A study by Deng et al [49] found an association between CV and diabetic macroangiopathy in people who were Hp1 carriers but not in people with the Hp2‐2 genotype. Furthermore, no studies found evidence for associations between macrovascular complications and the following CGM-derived metrics: CV, SD, TIR, TAR, glucose management indicator, time in tight range (TITR), lowest blood glucose value, and area under the TBR curve [27,47,48,50].

### Coronary Artery Disease

Six studies investigated coronary artery disease [28,40,43,46,48,53]. Studies reported an association between MAGE ≥3.4 mmol/L and coronary artery disease [28,43]. Furthermore, a difference was observed between the control group and the high blood glucose fluctuation group based on dichotomizing the following CGM metrics: SD <1.40 mmol/L, MAGE <3.90 mmol/L, largest amplitude of glycemic excursions <4.40 mmol/L, and mean of daily differences <0.83 mmol/L [28,43,53]. TIR <20% was found to have an association, but none of the other TIR intervals [53] or TITR [48] had an association. Lastly, hypoglycemic events were found to have an association with myocardial infarction but not with unstable angina pectoris [46]. Four studies investigated the severity of coronary artery disease [55] by using the Gensini score [28,40,43,54]. Three studies found an association with MAGE [28,43,54]. Further associations were found when comparing the control group with the high blood glucose fluctuation group [28,40]. Two studies investigated the complexity of coronary artery disease [56] using the SYNTAX score [30,54]. MAGE was found to have an association with the SYNTAX score [30,54] together with blood glucose excursions during the night from 00:00 to 03:00, in the mornings from 06:00 to 08:00, and at midday from 11:00 to 13:00. No association was detected for all the other times during the day [30].

### Stroke

Three studies investigated stroke or cerebrovascular accidents. TIR, but not hypoglycemic events, was associated with stroke [46,51]. TIR and TITR were both associated with cerebrovascular accidents [48].

### Peripheral Artery Disease

A study by De Meulemeester et al [48] included peripheral artery disease, while a study by Li et al [52] included lower extremity artery disease. Overall, they investigated CV, SD, TIR, and TITR, and only an association with TIR was found in some analyses [52].

## Discussion

### Main Findings and Methodological Considerations

This scoping review identified 53 studies focusing on the relationship between CGM-derived metrics and CVD risk in individuals with diabetes. The literature included inconsistent findings across association studies, which also had highly diverse clinical and subclinical CVD outcomes. CGM-derived metrics are widely studied, but their predictive value for CVD outcomes remains unclear since MAGE was the only metric whose predictive value was tested.

We observed patterns regarding study population size, diabetes type, and reporting of evidence for associations between CGM-derived metrics and CVD outcomes, as studies focusing on type 1 diabetes were often conducted in smaller study populations and rarely found evidence for associations. In contrast, studies focusing on type 2 diabetes were conducted in larger study populations and more consistently found evidence for associations. These patterns may explain some of the inconsistent findings for each CGM-derived metric and could indicate a lack of statistical power in some studies, suggesting that future studies, particularly those focusing on type 1 diabetes, should emphasize having sufficiently sized study populations. However, differences between type 1 and type 2 diabetes populations extend beyond sample size and include distinct pathophysiology, treatment regimens, and cumulative exposure to cardiovascular risk factors, all of which may influence the relationship between CGM-derived metrics and cardiovascular outcomes.

The most frequently investigated CGM-derived metric in our review was TIR, and we found inconsistent results across the included studies. Similar results were reported in the review by Yapanis et al [14]. The authors argued that low TIR is a risk factor for macrovascular disease and mentioned that the large sample size of a supporting study [21] provides more reliable evidence than the inconsistent results reported from smaller sample sizes. The same study [21] was the largest in our review, and its size makes its conclusion compelling. The inconsistency across other TIR studies is likely due to limited power and cross-sectional designs. MAGE was the CGM-derived metric most consistently associated with cardiovascular outcomes across studies and the only CGM-derived metric used for prediction; however, it exhibited poor discriminative ability. The more consistent associations observed for MAGE across studies, despite generally smaller sample sizes compared to TIR, may suggest a stronger link between MAGE and CVD risk than between TIR and CVD risk. Even though MAGE was studied across a diverse range of subclinical outcomes (Multimedia Appendix 7), studies on clinical outcomes were limited to coronary artery disease and severity scores, and studies on other clinical outcomes are needed to confirm this pattern, which would suggest that within-day glycemic variability may be an important cardiovascular risk factor. However, since MAGE is biased toward detecting hyperglycemic excursions [57], it may underestimate the impact of hypoglycemia on CVD. Although some studies have reported associations between hyperglycemia-focused CGM-derived metrics and cardiovascular outcomes [26,43], other studies have reported associations between hypoglycemia and cardiovascular outcomes [27,39,46,58-60], with some concluding that hypoglycemia is associated with macrovascular complications and hyperglycemia is associated with microvascular complications [27,59].

The heterogeneity in the reported associations between CGM-derived metrics and CVD outcomes may also reflect differences in how the metrics were defined and analyzed. Thresholds for TIR, TAR, and TBR, as well as observation periods, varied across studies. Statistical adjustment strategies also differed. Some studies adjusted for HbA_1c_ or other covariates, while others made no adjustments. However, we found no substantial differences between the unadjusted and the most adjusted estimates in the studies, suggesting that attenuation due to adjustment for other covariates played only a minor role. Study comparisons would have been easier if the analysis code were available; however, none of the authors provided this information. Together, these discrepancies highlight how diverse definitions and analytic approaches can contribute to conflicting findings and complicate the interpretation of the evidence in this field. The heterogeneity in this field makes it impossible to present findings in a quantitative meta-analysis, and there is a need for more standardized study designs if studies cannot generate definitive evidence by themselves.

Several recurring methodological issues also emerged. First, most studies were geographically concentrated in Asia and Europe, limiting generalizability to other health care settings and populations, particularly those in South America and Africa. Second, many studies assessed multiple combinations of CGM metrics and CVD outcomes in separate models without prespecified hypotheses or correction for multiple testing. Third, and most importantly, only 3 studies performed prediction modeling analyses, all of which were carried out as secondary analyses. None of the studies reported any external validation or performance metrics beyond discrimination (AUC, sensitivity, and specificity), indicating modest performance [61]. No study applied machine learning methods or used raw CGM time-series data, which may further constrain predictive ability. Thus, the predictive utility of CGM-derived metrics for CVD outcomes remains essentially untested. There is a clear need for sufficiently powered, longitudinal prediction studies using clinical CVD outcomes in ethnically diverse populations [62].

The vast majority of identified studies were cross-sectional, limiting their clinical relevance due to potential reverse causality, as established CVD can alter lifestyle behaviors and glucose patterns. This bias can skew the results in 2 directions. First, it may produce false-positive associations if distinct CGM patterns only emerge after a CVD event. Second, it can yield false-negative findings if incident CVD, or the resulting intensive medical treatment, masks or attenuates a pre-existing glucose pattern. However, there were too few longitudinal studies to assess if reverse causality systematically skewed the estimates provided by cross-sectional studies and thereby led to divergent results between the 2 study designs.

Collectively, the methodological challenges identified in this review indicate a need for clearer methodological alignment in future studies if systematic reviews are to be feasible. Specifically, researchers should adhere to consensus guidelines, such as the ATTD (Advanced Technologies and Treatments for Diabetes) consensus recommendations [63], together with prespecified covariate adjustment strategies, standardized classification of cardiovascular outcomes, and transparent reporting of analytic decisions. Addressing these areas would improve comparability across studies and strengthen the interpretability of future evidence.

Most studies used CGM data collected specifically for research, with only a few studies drawing on routinely collected real-world data despite the growing prevalence of CGM use. This represents a missed opportunity, as routine data are typically larger, more cost-effective, and more representative of CGM users. Underuse may reflect challenges in accessing data stored on proprietary manufacturer platforms or linking these data to individual health records. Open, publicly available datasets have driven advances in many fields (eg, medical image analysis) [64-66], but no comparable dataset exists for studying CGM data and complications. In the absence of such resources, aligning existing databases with FAIR (findability, accessibility, interoperability, and reusability) principles could help accelerate research in this area [67].

### Strengths and Limitations

A key strength of this review was the differentiation between association and prediction studies, highlighting the lack of knowledge on how well CGM-derived metrics perform in CVD prediction models. We performed a more comprehensive literature search, yielding an additional 40 studies compared to a previous review [14]. The distinction between clinical and subclinical CVD allowed a more detailed synthesis of how CGM-derived metrics relate to both CVD manifestation and early vascular changes. Furthermore, we provided a detailed methodological overview and revealed common methodological weaknesses, including variations in the calculation of CGM-derived metrics and the definitions of cardiovascular outcomes.

This review also has limitations. First, the feasibility of synthesizing effect sizes consistently across studies was limited by heterogeneity in study designs, CGM metrics, and CVD outcome definitions. Therefore, this review summarized studies based on *P* values, which is suboptimal, as *P* values are influenced by both the effect size and the sample size [68]. *P* values do not accurately reflect the effect size, clinical relevance, or estimate precision. This greatly limits our ability to compare the strength of associations across studies. Furthermore, underpowered studies are more likely not to find evidence for associations, thereby adding noise to the literature. Second, we reported only the most adjusted models from each study. While this approach was deemed necessary, it may have excluded potentially informative results from alternative model specifications. Third, identifying all relevant studies in this field proved challenging. We decided to limit the search to MEDLINE and Embase only, as these are core databases for biomedical literature searching. Given the resources available to the review team, we were not able to extend the database search further. However, the search retrieved a high number of records, both relevant and irrelevant, owing to inconsistent terminology and overlapping search categories (eg, “blood glucose monitoring” and “glycemic control” both encompass finger-prick measurements). We therefore designed a broad search strategy to ensure that we did not miss any relevant studies in the 2 databases that we chose to search. Acknowledging that searching only 2 databases may have resulted in missing relevant studies, we systematically screened all references and citing articles (backward and forward citation searching) of the included studies. This process resulted in the identification of 2 additional articles [44,69], of which 1 article (Koroleva et al [44]) was not indexed in the medical databases we searched. Nevertheless, to the best of our knowledge, this comprehensive search strategy enabled us to identify more relevant studies than any previous review on this topic.

### Conclusion

This scoping review mapped a broad landscape of association studies examining associations between CGM-derived metrics and CVD outcomes, with a smaller number also addressing prediction. The included studies were methodologically heterogeneous, making it difficult to synthesize evidence and draw firm conclusions about clinical cardiovascular risk.

Within these constraints and using statistical significance as a pragmatic indicator of consistency across heterogeneous studies covering different CVD outcomes, TIR was associated with CVD in the largest single study, and MAGE was the CGM-derived metric most consistently associated with CVD outcomes across multiple studies covering subclinical outcomes, coronary artery disease, and severity scores. Notably, MAGE was the only CGM-derived metric to have its predictive value assessed, and it exhibited only modest discriminatory performance. None of the studies used any machine learning–based methods, suggesting that the predictive value of CGM-derived metrics for CVD outcomes and the possibilities of using machine learning–based methods are underexplored. There is a fragmented evidence base in which metric definitions, study designs, and analytical strategies vary widely. In the future, more standardized analytical strategies could enable meta-analyses across individual studies to synthesize more substantial evidence.

## Supplementary material

10.2196/89374 Multimedia Appendix 1 Search string.

10.2196/89374 Multimedia Appendix 2 Subclinical cardiovascular outcome results.

10.2196/89374 Multimedia Appendix 3 List of continuous glucose monitoring metrics and cardiovascular disease outcomes found in the literature.

10.2196/89374 Multimedia Appendix 4 Included studies (with reference details) and their aims.

10.2196/89374 Multimedia Appendix 5 Studies excluded during full-text screening.

10.2196/89374 Multimedia Appendix 6 Geographical location of the studies.

10.2196/89374 Multimedia Appendix 7 Overview of the extracted results.

10.2196/89374 Multimedia Appendix 8 List of groupings of adjusted variables.

10.2196/89374 Multimedia Appendix 9 Summary of continuous glucose monitoring–derived metrics in each study and cardiovascular disease outcomes.

10.2196/89374 Multimedia Appendix 10 Full table (with adjustments) of the main findings of the included studies on clinical cardiovascular outcomes.

10.2196/89374 Checklist 1 PRISMA-ScR checklist.

## Abbreviations

AUC: area under the receiver operating characteristic curve
CGM: continuous glucose monitoring
CV: coefficient of variation
CVD: cardiovascular disease
HbA_1c_: hemoglobin A_1c_
MAGE: mean amplitude of glycemic excursions
PRISMA-ScR: Preferred Reporting Items for Systematic Reviews and Meta-Analyses extension for Scoping Reviews
TAR: time above range
TBR: time below range
TIR: time in range
TITR: time in tight range
