# Supplementary material for: Continuous Glucose Monitoring–Derived Metrics and Cardiovascular Risk Among People With Diabetes: Systematic Scoping Review
Source: JMIR Diabetes. 2026 May 6;11:e89374. doi: 10.2196/89374 (PMC13148326; doi:10.2196/89374)
Supplement: Multimedia Appendix 1 [file diabetes-v11-e89374-s001.docx]

# Multimedia Appendix 1

| **Table S1: Search in Ovid MEDLINE** | | |
| --- | --- | --- |
| Search date: 22 January 2024 | | |
| **#** | **Searches** | **Results** |
| 1 | Blood Glucose Self-Monitoring/ | 10241 |
| 2 | Continuous Glucose Monitoring/ | 34 |
| 3 | (((blood-glucose or bloodglucose or glucose) adj2 (monitor$ or selfmonitor$)) or cgm or bgm or iscgm or rtcgm or (time adj3 range)).ti,ab,kf,kw. | 28749 |
| 4 | (Blood Glucose/ or Glycemic Control/ or (bloodglucose$ or bloodsugar$ or glucose$ or glyc?emic$ or sugar$).ti,ab,kf,kw.) adj5 (flash or fluctuat$ or intermittent$ or scan$ or variabilit$).ti,ab,kf,kw. | 8669 |
| 5 | or/1-4 | 38366 |
| 6 | exp Cardiovascular Diseases/ | 2760764 |
| 7 | (cardiovasc$ or cvd or cardiometabol$ or cardio-metabol$ or heart$ or macrovasc$ or vasc$).ti,ab,kw,kf. | 2182473 |
| 8 | or/6-7 | 3971342 |
| 9 | exp Diabetes Mellitus/ | 518864 |
| 10 | (diabet$ or dm1 or dm2 or dmi or dmii or iddm or niddm or t1d or t2d).ti,ab,kw,kf. | 815092 |
| 11 | or/9-10 | 875527 |
| 12 | and/5,8,11 | 2416 |
| 13 | exp Animals/ not Humans/ | 5189493 |
| 14 | 12 not 13 | 2300 |
| Updated search: 11 March 2025 | | |
| 15 | limit 14 to (da="20231201-20261231" or dt="20231201-20261231" or ez="20231201-20261231" or ed="20231201-20261231") | 310 |
| 16 | or/14-15 | **2610** |

| **Table S2: Search in Embase (Ovid)** | | |
| --- | --- | --- |
| Search date: 22 January 2024 | | |
| **#** | **Searches** | **Results** |
| 1 | blood glucose monitoring/ | 37574 |
| 2 | exp continuous glucose monitoring system/ | 5648 |
| 3 | (((blood-glucose or bloodglucose or glucose) adj2 (monitor$ or selfmonitor$)) or cgm or bgm or iscgm or rtcgm or (time adj3 range)).ti,ab,kf,kw. | 48502 |
| 4 | (exp glucose blood level/ or glycemic control/ or (bloodglucose$ or bloodsugar$ or glucose$ or glyc?emic$ or sugar$).ti,ab,kf,kw.) adj5 (flash or fluctuat$ or intermittent$ or scan$ or variabilit$).ti,ab,kf,kw. | 16430 |
| 5 | or/1-4 | 81510 |
| 6 | exp cardiovascular disease/ | 5122163 |
| 7 | (cardiovasc$ or cvd or cardiometabol$ or cardio-metabol$ or heart$ or macrovasc$ or vasc$).ti,ab,kw,kf. | 3044925 |
| 8 | or/6-7 | 6303633 |
| 9 | exp diabetes mellitus/ | 1239184 |
| 10 | (diabet$ or dm1 or dm2 or dmi or dmii or iddm or niddm or t1d or t2d).ti,ab,kw,kf. | 1237019 |
| 11 | or/9-10 | 1501812 |
| 12 | and/5,8,11 | 11217 |
| 13 | (rat or rats or mouse or mice or swine or porcine or murine or sheep or lambs or pigs or piglets or rabbit or rabbits or cat or cats or dog or dogs or cattle or bovine or monkey or monkeys or trout or marmoset$1).ti. and animal experiment/ | 1237966 |
| 14 | animal experiment/ not (human experiment/ or human/) | 2601109 |
| 15 | or/13-14 | 2672240 |
| 16 | 12 not 15 | 10861 |
| 17 | limit 16 to exclude medline journals | 2176 |
| Updated search: 11 March 2025 | | |
| 18 | limit 17 to (dc="20231201-20261231" or dd="20231201-20261231" or rd="20231201-20261231") | 467 |
| 19 | or/17-18 | **2643** |
