# Supplementary material for: Continuous Glucose Monitoring–Derived Metrics and Cardiovascular Risk Among People With Diabetes: Systematic Scoping Review
Source: JMIR Diabetes. 2026 May 6;11:e89374. doi: 10.2196/89374 (PMC13148326; doi:10.2196/89374)
Supplement: Multimedia Appendix 2 [file diabetes-v11-e89374-s002.docx]

**Multimedia Appendix 2**

# Subclinical Cardiovascular Outcomes

Table S1 summarizes the main findings of each study on subclinical cardiovascular outcomes.

**Table S1: Study design, demographics, and adjustments of the included subclinical cardiovascular outcome studies**

| **Author, year** | **Study Design** | **Population + age [years]** | **Size of popu-**  **lation** | **CGM duration** | **Adjusted** |
| --- | --- | --- | --- | --- | --- |
| Chen, 2020†^1^ | Longitudinal FU: within 3 months | T2D + CAD,  Only male  Age: 45-70 | 210 | 3rd and 5th day of the study | Univariate |
| Mita, 2023^2^ | Longitudinal FU: 104 weeks | T2D Age: 30-80 | 553 | 14 days at baseline and at week 104 | Demographic Anthropometric Lifestyle Medical History Physiological Biochemical Medication CVD marker |
| Bergdahl, 2025^3^ | Cross-sectional | T1D, n = 50 Normal controls, n = 41 Age 6-15.99 | 91 |  | Demographic Anthropometric Physiological Biochemical CGM/GV metrics |
| Borg, 2011^4^ | Cross-sectional | T1D = 268 T2D = 159  Age: 18-70 | 427 | 48 hours *3 times | Demographic Lifestyle Medical History |
| Buscemi, 2010^5^ | Cross-sectional | Metabolic syndrome = 23 Withhout metabolic syndrome = 30 T2D not diagnosed prior to study = 22  Age: 30-65 | 75 | 46-48 hours | nr |
| Cai, 2024^6^ | Cross-sectional | T2D Age ≥ 18 year | 342 | 14 days | Demographic Anthropometric Lifestyle Medical History Physiological Biochemical |
| Castaldo, 2011^7^ | Cross-sectional | Normal glucose tolerance = 36 Impaired glucose tolerance = 43  Age: 23–70 | 79 | 72 hours | Demographic Anthropometric Lifestyle Physiological Biochemical CGM/GV metrics |
| Cesana, 2013^8^ | Cross-sectional | T1D, free of CVD Mean age±SD: 40.8 ± 7.6 | 17 | 24 hours | Univariate |
| Chen, 2010^9^ | Cross-sectional | T2D with  CVD = 16 T2D without  CVD = 20 Normal  controls = 10  Age: 33–86 | 46 | 72 hours | Univariate |
| Cutruzzola, 2022^10^ | Cross-sectional | T1D = 70 Mean age: 35 ± 13   Controls, n = 35 comparable on sex and age Mean age±SD: 32 ± 13 | 105 | 6 months | nr |
| De Meulemeester, 2024†^11^ | Cross-sectional | T1D  Mean age±SD: 44.8 ± 15.2 | 808 | 2 weeks | Demographic Anthropometric Lifestyle Medical History Biochemical Medication |
| Di Flaviani, 2011^12^ | Cross-sectional | T2D Mean age±SD: 59.2 ± 10.6 | 26 | 24 hours | Demographic Physiological Biochemical CGM/GV metrics CVD marker |
| Dzhun, 2023^13^ | Cross-sectional | T2D without coronary artery disease Mean age±SD: 47.5 ± 4 | 78 | 6 days | Demographic Anthropometric Medical History Physiological Biochemical CVD marker |
| Foreman, 2021^14^ | Cross-sectional | Euglycemia, n = 454 Prediabetes, n = 175 T1D, n = 2 T2D, n = 185 Age: 40-75 | 643-816 | 7 days | Demographic Anthropometric Lifestyle Physiological Biochemical Medication CGM/GV metrics |
| Georeli, 2024^15^ | Cross-sectional | T1D Age: 4-21 | 124 | 24 hours | Demographic  Anthropometric |
| Gimenez, 2011^16^ | Cross-sectional | T1D = 38 Normal controls = 22 Age- and sex-matched Age > 18 years | 60 | 72 hours | Univariate |
| Gordin, 2008^17^ | Cross-sectional | T1D, males only  Age: 25.9 ± 5.6 | 22 | 72 hours | ct |
| Helleputte, 2022^18^ | Cross-sectional | T1D free of CVD Age > 18 | 54 | 7 days | Univariate |
| Hoffman, 2013^19^ | Cross-sectional | T1D Age: 13.1 ± 1.6 | 17 | 3 days | Univariate |
| Kakuta, 2017^20^ | Cross-sectional | T2D with coronary artery disease or suspected coronary artery disease Age >20 | 71 | 7 days | nr |
| Koroleva, 2022^21^ | Cross-sectional | T2D on antihyperglycemic therapy Age>40 | 194 | 3 days | Demographic Medical History Biochemical |
| Lu, 2020^22^ | Cross-sectional | T2D Age ≥ 18 | 2215 | 3 days | Demographic Anthropometric Lifestyle Medical History Physiological Biochemical Medication |
| Magri, 2018†^23^ | Cross-sectional | T2D Median age (IQR): 64 (57–68) | 121 | 72 hours | Anthropometric Lifestyle Biochemical CGM/GV metrics |
| Mesa, 2022^24^ | Cross-sectional | T1D Age ≥ 40 | 152 | 14 days | Demographic Medical History Physiological Biochemical Medication |
| Mo, 2013^25^ | Cross-sectional | T2D Age: 40-86 | 216 | 72 hours | Demographic Anthropometric Lifestyle Medical History Physiological Biochemical Medication CGM/GV metrics |
| Morandi, 2023^26^ | Cross-sectional | T1D Age: 9-23 | 267 | 4 weeks | nr |
| Pena, 2012^27^ | Cross-sectional | T1D =52 Mean age±SD: 14±2.7 Normal controls = 50 (Age and sex matched) Mean age(SD): 14.8±3.3 | 102 | 4 days Days 0 and 3 where not used (Results in 48 hours used for calculations) | Anthropometric Medical History |
| Pertseva 2023^28^ | Cross-sectional | T2D, n = 53 Normal control, n = 10 Age, sex and BMI matched Age: 51-65 | 63 | 24 hours | Univariate |
| Piona, 2023^29^ | Cross-sectional | T1D Age: 2-18 years | 895 | 2 weeks previsit and 4 weeks previsit | Demographic Anthropometric Medical History Biochemical Medication CGM/GV metrics |
| Pulkkinen, 2020^30^ | Cross-sectional | T1D Age:12-15.9 | 39 | 6 days for baseline + 12 months | Univariate |
| Snell, Bergeon, 2010^31^ | Cross-sectional | T1D, no history of coronary artery disease Age 19-56 years | 75 | 5 days | Demographic Medical History CGM/GV metrics CVD marker |
| Taya, 2021^32^ | Cross-sectional | T2D , no history of CVD age 30-80 | 600 | 3-10 days | Demographic Anthropometric Lifestyle Medical History Physiological Biochemical |
| Torimoto, 2025^33^ | Cross-sectional | T2D no history of CVD Age: 30–80 | 999 |  | Demographic Anthropometric Lifestyle Medical History Physiological Biochemical Medication |
| Wakasugi, 2021^34^ | Cross-sectional | T2D, no history of CVD Age 30-80 | 445 | 14 days | Demographic Anthropometric Lifestyle Medical History Physiological Biochemical Medication |
| Wei, 2022^35^ | Cross-sectional | T2D Median age(IQR): 55.0 (47 - 65) | 405 | 72 hours | Demographic Anthropometric Medical History Physiological Biochemical CGM/GV metrics |
| Yano, 2013^36^ | Cross-sectional | T2D Age ≥ 20 | 49 | 72 hours | Physiological |
| Yokota, 2019^37^ | Cross-sectional | T2D, free of CVD Mean age±: 60 ± 14 | 100 | 72 hours | Demographic Anthropometric Medical History Biochemical Medication CVD marker |
| Zhang, 2014^38^ | Cross-sectional | T2D - With CAD, n=52 - Without CAD, n = 36 Healthy controls, n=30 Age: 50-70 | 118 | 72 hours | Physiological Biochemical |
| Zhang, 2020^39^ | Cross-sectional | T2D Age: 25-75 | 78 | 72 hours | Univariate |
| Zhou, 2022^40^ | Cross-sectional | T2D, hospitalized, no history of CVD Median age(IQR): 55 (47,64) | 469 | 72 hours | Demographic Anthropometric Medical History Physiological Biochemical |

Age is reported in years either as an interval, mean±SD or median(IQR).

Abbreviations: CGM, continuous glucose monitoring; FU, follow-up; T1D, type 1 diabetes; T2D, type 2 diabetes; CAD, coronary artery disease; CVD, cardiovascular disases; BMI, body mass index; GV, glycemic variability; nr, not reported; ct, cannot tell.

**Table S2: Outcomes of the included subclinical cardiovascular outcome studies**

|  | **Author, year** | **Outcomes** | **MBG** | **CV** | **SD** | **TIR** | **TBR** | **TAR** | **MAGE** | **MODD** | **Other** |
| --- | --- | --- | --- | --- | --- | --- | --- | --- | --- | --- | --- |
| **Arterial stiffness** | Morandi, 2023*^26^ | cf-PWV | ❌ |  | ❌ | ❌ | ❌ | ❌ | ❌ |  | CONGA ❌ |
|  | Helleputte, 2022^18^ | cf-PWV |  | ❌ | ❌ | ❌ | ❌ | ❌ | ❌ | ❌ |  |
|  | Foreman, 2021*^14^ | cf-PWV |  | ✅ | ❌ | ✅ |  |  |  |  |  |
|  | Pulkkinen, 2020*^30^ | cf-PWV | ❌ | ❌ | ❌ | ❌ |  |  |  |  |  |
|  |  | cr-PWV | ❌ | ❌ | ❌ | ✅ |  |  |  |  |  |
|  | Torimoto, 2025*^33^ | ba-PWV |  |  |  |  |  |  |  |  | GRI ✅ |
|  | Cai, 2024^6^ | ba-PWV |  | ✅ |  | ✅ |  |  | ✅ |  | GRI ✅❌ |
|  | Zhou, 2022^40^ | High ba-PWV ≥ 1800 cm/s Low ba-PWV |  |  |  | ✅ |  |  |  |  |  |
|  | Wakasugi, 2021^34^ | ba-PWV | ✅ | ✅ | ✅ | ✅ | ✅ | ✅ | ✅ | ❌ | HBGI ✅ LBGI ❌ IQR ❌ |
|  |  | High ba-PWV ≥ 1800 cm/s | ❌ | ❌ | ✅ | ❌ | ❌ | ✅ ❌ | ✅ | ❌ | HBGI ✅ LBGI ❌ IQR ❌ |
|  | Yano, 2013*^36^ | ba-PWV | ✅ |  |  |  |  |  |  |  | PPGE ✅ |
|  | Georeli, 2024^15^ | PWV |  |  |  | ✅❌ | ❌ | ❌ |  |  |  |
|  | Gordin, 2008*^17^ | Aortic PWV | ✅ |  |  |  |  |  | ✅ |  |  |
|  | Gordin, 2008*^17^ | Augmentation index | ✅ |  |  |  |  |  | ✅ |  |  |
|  | Cesana, 2013*^8^ | Carotid artery distensibility | ❌ |  | ❌ |  |  |  | ❌ |  |  |
|  | Foreman, 2021*^14^ | Carotid distensibility coefficient |  | ❌ | ❌ | ✅ |  |  |  |  |  |
| **Flow resistance** | Cutruzzola, 2022*^66^ | FMD | ❌ | ❌ |  | ❌ | ❌ | ❌ |  |  |  |
|  | Zhang, 2014^38^ | FMD |  |  |  |  |  |  | ✅ |  |  |
|  | Pena, 2012^27^ | FMD |  |  |  |  | ❌ |  |  |  | Hypoglycemia ✅ LBGI ✅ |
|  | Gimenez, 2011*^16^ | FMD |  |  |  |  |  |  | ✅ |  |  |
|  | Buscemi, 2010*^5^ | FMD | ✅ | ✅ |  |  |  |  | ❌ |  | AUC ✅ |
|  | Foreman, 2021*^14^ | ABI |  | ❌ | ❌ | ✅ ❌ |  |  |  |  |  |
|  | Wei, 2022^35^ | ABI ≤ 0.9 ABI ≥ 1.3 | ❌ | ❌ | ❌ | ✅ |  | ❌ | ❌ | ❌ | M value ✅❌ ADRR ✅ |
|  | Zhang, 2020^39^ | ABI | ✅ |  | ✅ |  | ❌ | ✅ | ✅ | ❌ | LAGE ✅ |
|  | Hoffman, 2013^19^ | Forearm vascular resistance | ✅ |  |  |  |  |  |  |  |  |
|  |  | Forearm Blood Flow | ❌ |  |  |  |  |  |  |  |  |
| **Arterial wall thickness** | Bergdahl, 2025^3^ | intima thickness, media thickness, and IMT of the radial, dorsal pedal, and carotid arteries. |  | ❌ |  |  |  |  |  |  | TITR ✅ ❌ |
|  | Pulkkinen, 2020*^30^ | Femoral IMT | ❌ | ❌ | ❌ | ❌ |  |  |  |  |  |
|  | Lu, 2020*^22^ | CCA-IMT |  | ❌ | ❌ | ❌ |  |  | ❌ |  |  |
|  | Yano, 2013*^36^ | CCA-IMT | ✅ |  |  |  |  |  |  |  |  |
|  | Taya, 2021*^32^ | CCA-max-IMT | ❌ | ❌ | ❌ | ❌ | ❌ | ❌ | ❌ | ❌ | Median ❌ IQR ❌ HBGI ❌ LBGI ❌ |
|  | Mita, 2023*^2^ | CCA-max-IMT change | ❌ | ❌ |  | ❌ | ❌ | ❌ |  |  |  |
|  | Torimoto, 2025*^33^ | mean CIMT |  |  |  |  |  |  |  |  | GRI ✅ |
|  | Mita, 2023*^2^ | Mean CIMT change | ❌ | ❌ |  | ❌ | ❌ | ❌ |  |  |  |
|  | Morandi, 2023*^26^ | CIMT | ❌ |  | ❌ | ❌ | ❌ | ❌ | ❌ |  | CONGA ❌ |
|  | Cutruzzola, 2022*^10^ | CIMT | ❌ | ❌ |  | ❌ | ❌ | ❌ |  |  |  |
|  | Koroleva, 2022*^21^ | CIMT/plaques |  |  |  |  |  |  | ✅ |  |  |
|  | Foreman, 2021*^14^ | CIMT |  | ❌ | ❌ | ❌ |  |  |  |  |  |
|  | Taya, 2021*^32^ | CIMT | ❌ | ❌ | ❌ | ❌ | ❌ | ❌ | ❌ | ❌ | Median ❌ IQR ❌ HBGI ❌ LBGI ❌ |
|  | Lu, 2020*^22^ | Mean CIMT |  | ❌ | ❌ | ❌ |  |  | ❌ |  |  |
|  |  | Abnormal CIMT: CIMT ≥1.0 mm |  | ❌ | ❌ | ✅ |  |  | ❌ |  |  |
|  | Pulkkinen, 2020*^30^ | CIMT | ❌ | ❌ | ❌ | ❌ |  |  |  |  |  |
|  | Magri, 2018†^23^ | CIMT (Abnormal) | ❌ |  | ❌ | ❌ | ✅ | ❌ |  |  | AUC ❌ min. value ❌ max. value ❌ Number of highest excursions ❌ Number of lowest excursions ❌ |
|  | Cesana, 2013*^8^ | CIMT | ❌ |  | ❌ |  |  |  | ❌ |  |  |
|  | Mo, 2013^25^ | CIMT | ✅ |  | ❌ |  |  |  | ✅ |  |  |
|  | Castaldo, 2011^7^ | CIMT | ❌ | ❌ |  |  |  |  |  |  | Duration in hypoglycemia [min/day] ✅ |
|  | Gimenez, 2011*^16^ | CIMT |  |  |  |  |  |  | ✅ |  |  |
|  | Buscemi, 2010*^5^ | CIMT | ❌ | ❌ |  |  |  |  | ❌ |  | AUC ❌ |
|  | Chen, 2010^9^ | CIMT |  |  |  |  |  |  | ✅ |  | LAGE ✅ |
| **Arterial wall composition** | Torimoto, 2025*^33^ | Mean GSM |  |  |  |  |  |  |  |  | GRI ❌ |
|  | Mita, 2023^2^ | Mean GSM | ❌ | ❌ |  | ❌ | ❌ | ❌ |  |  |  |
|  |  | Thickened GSM change | ❌ | ✅ |  | ✅ | ❌ | ❌ |  |  |  |
|  | Taya, 2021*^32^ | Mean GSM | ✅ | ✅ | ✅ | ✅ | ❌ | ✅ ❌ | ✅ | ✅ | Median ❌ IQR ❌ HBGI ✅ LBGI ❌ |
|  | Mesa, 2022^24^ | Carotid plaque | ❌ | ❌ |  | ❌ | ✅ | ❌ |  |  | GMI ❌ |
|  | Snell Bergeon, 2010^31^ | Coronary artery calcification | ✅ |  | ✅ |  | ❌ | ✅ |  |  | time outside of target range ✅ |
|  | Kakuta, 2017^20^ | lipidic volume (%) fibrotic volume (%) percent necrotic volume (%) percent calcified volume (%) ... in coronary plaques | ❌ |  |  |  | ❌ | ❌ | ✅ ❌ |  |  |
| **Cardiac and pulse related measures** | Foreman, 2021*^14^ | Mean circumferential wall stress |  | ❌ | ❌ | ❌ |  |  |  |  |  |
|  |  | Pulsatile circumferential wall stress |  | ❌ | ❌ | ❌ |  |  |  |  |  |
|  | Borg, 2011^4^ | - CVD risk factor Z-score | ✅ |  | ❌ |  |  |  | ❌ |  | AUC ✅ PPGE ❌ CONGA ❌ |
|  | Chen, 2020†^1^ | Statistical difference between normal BG group and high BG fluctuation group on all Heartrate variability parameters |  |  |  |  |  |  |  |  | High and low BG fluctuations by own definition using SD, MAGE, MODD and LAGE ✅ |
|  | Dzhun, 2023^13^ | SD ≥ 2 -> Left Ventricular Diastylic Dysfunction |  |  | ❌ |  |  |  |  |  |  |
|  | Di Flaviani, 2011^12^ | Left ventricular mass index |  |  |  |  |  |  |  |  | CONGA ❌ |
|  | Yokota, 2019^37^ | Left ventricular diastolic function |  |  | ✅ |  |  |  |  |  |  |
|  | Yano, 2013*^36^ | Left ventricular mass index | ❌ |  |  |  |  |  |  |  | PPGE ❌ |
|  | Pertseva, 2023^28^ | Average variability |  |  | ❌ | ❌ | ❌ | ❌ |  |  |  |
|  |  | Average Pulse blood pressure Pulse blood pressure variability |  |  | ❌ | ❌ | ❌ | ❌ |  |  |  |
|  |  | Average SBP,SBP variability,, |  |  | ✅ | ✅ | ✅ | ✅ |  |  |  |
|  |  | Average DBP DBP variability |  |  | ✅ | ✅ | ✅ | ✅ |  |  |  |
|  | Piona, 2023^29^ | Systolic and diastolic blood pressure | ❌ | ❌ | ❌ | ❌ | ❌ | ❌ |  |  |  |
|  | Pulkkinen, 2020*^30^ | Systolic and diastolic blood pressure | ❌ | ❌ | ❌ | ❌ |  |  |  |  |  |
| **Arterial lumen** | De Meulemeester, 2024 †^11^ | Stenosis of the carotid artery |  |  |  | ❌ |  |  |  |  | TITR ❌ |
|  | Cutruzzola, 2022*^10^ | Baseline brachial artery ID (mm) | ❌ | ❌ |  | ❌ | ❌ | ❌ |  |  |  |
|  | Koroleva, 2022*^21^ | stenosis and/or occlusion |  |  |  |  |  |  | ❌ |  |  |

* This study appears multiple times in the clinical cardiovascular disease outcome tables.

✅ An association was found between the given CGM-derived metric and the CVD outcome.

❌ There was no association found between the given CGM-derived metric and the CVD outcome.

A ✅ and a ❌ in the same cell means that the study either have multiple CGM-derived metrics or CVD outcomes which where aggregated in this review.

Empty cells in this table means that the association between the given CGM derived metric and the CVD outcome was not investigated.

Further elaboration on the adjusted variables can be found in Additional files 6 and 7.

Abbreviations: PWV, pulse wave velocity; ba-PWV, brachial-ankle pulse wave velocity; crPWV, carotid-radial pulse wave velocity; cf-PWV = Carotid-femoral pulse wave velocity; FMD, flow mediated dilation; ABI, ankle-brachial index; IMT, intima media thickness; CCA-IMT, intima media thickness of the common carotid artery; CIMT, carotid artery intima-media thickness; GSM, grey-scale median; CVD, cardiovascular disease; SBP, systolic blood pressure; DBP, diastolic blood pressure; BG, blood glucose; MBG, mean blood glucose; CV, coefficient of variation; SD, standard deviation; TIR, time in range; TBR, time below range; TAR, time above range; MAGE, mean amplitude of glycemic excursions; MODD, mean of daily differences; CONGA, continuous overall net glycemic action; GRI, glycemic risk index; HBGI, high blood glucose index; LBGI, low blood glucose index; IQR, interquartile range; PPGE, postprandial glucose excursions; AUC, area under curve; ADRR, average daily risk range; LAGE, largest amplitude of glycemic excursions; TITR, time in tight range; GMI, glucose management index; min, minimum; max, maximum.

## Arterial Stiffness

The subclinical outcomes covering arterial stiffness included measurements of carotid-radial, carotid-femoral, brachial-ankle, and aortic pulse wave velocity, in addition to carotid artery distensibility, and the augmentation index of systemic arterial stiffness. The outcomes were covered across 12 studies. Eleven studies investigated associations between CGM-derived metrics and pulse wave velocity. The CGM-derived metrics were MBG, CV, SD, TIR, TBR, TAR, MAGE, MODD, glucose risk index (GRI), HBGI, LBGI, interquartile range (IQR), continuous overall net glycemic action (CONGA), and PPGE. No association was found between MODD and pulse wave velocity.^18,34^ The associations between the other CGM-derived metrics and pulse wave velocity were more contradictory. Some studies found associations with a few CGM-derived metrics,^14,34,36^ some studies found associations with all or almost all their CGM-derived metrics, ^6,17,33,34,40^ while others did not find any.^18,26^ One study by Gordin et al. ^17^ found associations between the CGM-derived metrics MBG and MAGE with the augmentation index. Only TIR had an association with carotid distensibility, even though MBG, SD, MAGE, CV, and SD were also tested. However, no other CGM-derived metrics were found to have an association with arterial stiffness outcomes.^8,14^ (Table S2)

## Flow Resistance

Flow resistance covered the measurements of flow-mediated dilation of the brachial artery, ankle–brachial index, forearm vascular resistance, and forearm blood flow.

In total, nine studies investigated flow resistance measurements. Five studies included flow-mediated dilation of the brachial artery with varying results. Some studies found associations with the following CGM-derived metrics: MBG CV, MAGE, area under the curve (AUC), hypoglycemic events, and LBGI,[45,66–68] whereas other studies did not find any associations with the CGM-derived metrics: MBG, CV, TIR, TBR, TAR, and MAGE.^5,10,27^ Three articles included the ankle-brachial index.^14,35,39^ There were associations between the CGM-derived metrics LAGE and ADRR with ankle-brachial index, uncertainties with TIR, MBG, SD, MAGE, M-value, and no associations between CV, TBR, and MODD. Lastly, the study by Hoffman et al. ^19^ found an association between MBG and forearm vascular resistance, but not with MBG and forearm blood flow. (Table S2)

## Arterial Wall Thickness

Eighteen studies included CVD outcomes related to arterial wall thickness, such as carotid intima-media thickness measurements. TITR, duration in hypoglycemia, GRI, and LAGE were found to be associated with intima-media thickness measurements.^7,9,33^ There were contradicting results regarding TIR, TBR, TITR, MAGE, and their associations with intima-media thickness measurements, since eight studies found some associations,^3,9,16,21–23,25,36^ while nine studies did not find any association between their CGM-derived metrics (MBG, CV, SD, TIR, TBR, TAR, MAGE, MODD, AUC, CONGA, HBGI, LBGI,  median, and IQR) and intima-media thickness measurements.^2,3,5,8,10,14,22,26,30,32^ The studies did agree that CV and TAR were not associated with intima-media thickness measurements. Furthermore, the lowest and highest blood glucose values, and the number of the highest and lowest excursions, were not associated with intima-media thickness measurements.^23,36^ (Table S2)

## Arterial Wall Composition

Six studies included measurements of arterial wall composition as outcomes, such as the presence of plaques, plaque composition, or Gray-scale median of the carotid arteries. Associations with coronary plaque composition were consistently found for SD and time outside of target range,^20,31,32^ whereas contradicting results were found for MBG, TBR, TAR and MAGE, ^20,24,31^ and no associations were reported between CV, TIR, GMI and plaque compositions.^24^ SD, MAGE, MODD, and HBGI were found to have an association with the gray-scale median of the carotid arteries.^32^ No associations were found between TBR, IQR, LBGI, GRI and the CVD outcome grey-scale median.^2,32,33^ At the same time, there were contradicting results for an association between the CGM-derived metrics MBG, CV, TIR, and TAR and the grey-scale median. (Table S2)

## Cardiac and Pulse-related Measures

Ten studies included outcomes related to the heart or pulse, covering left ventricular diastolic function, left ventricular mass index, mean circumferential wall stress, pulsatile circumferential wall stress, systolic blood pressure, diastolic blood pressure, pulse blood pressure, heart rate variability parameters, and CVD risk factor z-score. Two-hour postprandial AUC was found to be associated with some heart or pulse outcomes^1,4^ while CONGA, postprandial measurements, MAGE and CV were not.^12–14,29,30,36^ MBG, SD, TIR, TBR, and TAR, were found to be associated with pulse or heart outcomes in some studies, but not in others.^1,4,14,28–30,36,37^ (Table S2)

## Arterial Lumen

Three studies investigated the arterial lumen.^10,11,21^ While a study by Koroleva et al. [49] found MAGE ≥3.4 mmol/L to be a risk factor and predictor of stenosis and/or occlusion in the univariate AUC analysis described above, MAGE was removed from the adjusted analysis during variable selection. There was no relation found between stenosis and the CGM-derived metrics TIR or TITR^11^ or brachial artery internal diameter and the CGM-derived metrics: MBG, CV, SD, TIR, TBR, and TAR.^10^ (Table S2)

## References

1. Chen Y, Jia T, Yan X, et al. Blood glucose fluctuations in patients with coronary heart disease and diabetes mellitus correlates with heart rate variability: A retrospective analysis of 210 cases. Nigerian journal of clinical practice 2020;23(9):1194–1200; doi: 10.4103/njcp.njcp_529_19.

2. Mita Tomoya, Katakami Naoto, Okada Yosuke, et al. Continuous glucose monitoring-derived time in range and CV are associated with altered tissue characteristics of the carotid artery wall in people with type 2 diabetes. Diabetologia 2023;66(12):2356–2367; doi: 10.1007/s00125-023-06013-3.

3. Bergdahl E, Forsander G, Sundberg F, et al. Investigating the presence and detectability of structural peripheral arterial changes in children with well-regulated type 1 diabetes versus healthy controls using ultra-high frequency ultrasound: a single-centre cross-sectional and case-control study. eClinicalMedicine 2025;81:103097; doi: 10.1016/j.eclinm.2025.103097.

4. Borg R, Kuenen J C, Carstensen B, et al. HbA1(c) and mean blood glucose show stronger associations with cardiovascular disease risk factors than do postprandial glycaemia or glucose variability in persons with diabetes: the A1C-Derived Average Glucose (ADAG) study. ADAG Study Group. ed. Diabetologia 2011;54(1):69–72; doi: 10.1007/s00125-010-1918-2.

5. Buscemi S, Re A, Batsis J A, et al. Glycaemic variability using continuous glucose monitoring and endothelial function in the metabolic syndrome and in Type 2 diabetes. Comment in: Diabet Med 2011 Jan;28(1):125-6; author reply 127-8 PMID: 21166857 [https://www.ncbi.nlm.nih.gov/pubmed/21166857] Comment in: Diabet Med 2011 Jan;28(1):126; author reply 127-8 PMID: 21166858 [https://www.ncbi.nlm.nih.gov/pubmed/21166858] 2010;27(8):872–8; doi: 10.1111/j.1464-5491.2010.03059.x.

6. Cai L, Shen W, Li J, et al. Association between glycemia risk index and arterial stiffness in type 2 diabetes. J of Diabetes Invest 2024;15(5):614–622; doi: 10.1111/jdi.14153.

7. Castaldo Ersilia, Sabato Donata, Lauro Davide, et al. Hypoglycemia assessed by continuous glucose monitoring is associated with preclinical atherosclerosis in individuals with impaired glucose tolerance. PloS one 2011;6(12):e28312; doi: 10.1371/journal.pone.0028312.

8. Cesana Francesca, Giannattasio Cristina, Nava Stefano, et al. Impact of blood glucose variability on carotid artery intima media thickness and distensibility in type 1 diabetes mellitus. Blood pressure 2013;22(6):355–61; doi: 10.3109/08037051.2013.791413.

9. Chen Xiao-min, Zhang Yan, Shen Xing-ping, et al. Correlation between glucose fluctuations and carotid intima-media thickness in type 2 diabetes. Diabetes research and clinical practice 2010;90(1):95–9; doi: 10.1016/j.diabres.2010.05.004.

10. Cutruzzola Antonio, Parise Martina, Scavelli Faustina B, et al. Time in Range Does Not Associate With Carotid Artery Wall Thickness and Endothelial Function in Type 1 Diabetes. Journal of diabetes science and technology 2022;16(4):904–911; doi: 10.1177/1932296821993178.

11. De Meulemeester J, Charleer S, Visser MM, et al. The association of chronic complications with time in tight range and time in range in people with type 1 diabetes: a retrospective cross-sectional real-world study. Diabetologia 2024;67(8):1527–1535; doi: 10.1007/s00125-024-06171-y.

12. Di Flaviani Alessandra, Picconi Fabiana, Di Stefano Paola, et al. Impact of glycemic and blood pressure variability on surrogate measures of cardiovascular outcomes in type 2 diabetic patients. Diabetes care 2011;34(7):1605–9; doi: 10.2337/dc11-0034.

13. Dzhun Yana, Mankovsky Georgy, Rudenko Nadiya, et al. Glycemic variability is associated with diastolic dysfunction in patients with type 2 diabetes. Journal of diabetes and its complications 2023;37(11):108519; doi: 10.1016/j.jdiacomp.2023.108519.

14. Foreman Yuri D, van Doorn William P T M, Schaper Nicolaas C, et al. Greater daily glucose variability and lower time in range assessed with continuous glucose monitoring are associated with greater aortic stiffness: The Maastricht Study. Diabetologia 2021;64(8):1880–1892; doi: 10.1007/s00125-021-05474-8.

15. Georeli E, Stamati A, Dimitriadou M, et al. Assessment of arterial stiffness in paediatric patients with type 1 diabetes mellitus. Journal of Diabetes and its Complications 2024;38(8):108782; doi: 10.1016/j.jdiacomp.2024.108782.

16. Gimenez Marga, Gilabert Rosa, Lara Merce, et al. Preclinical arterial disease in patients with type 1 diabetes without other major cardiovascular risk factors or micro-/ macrovascular disease. Diabetes & vascular disease research 2011;8(1):5–11; doi: 10.1177/1479164110388674.

17. Gordin D, Ronnback M, Forsblom C, et al. Glucose variability, blood pressure and arterial stiffness in type 1 diabetes. Diabetes research and clinical practice 2008;80(3):e4-7; doi: 10.1016/j.diabres.2008.01.010.

18. Helleputte Simon, Calders Patrick, Rodenbach Arthur, et al. Time-varying parameters of glycemic control and glycation in relation to arterial stiffness in patients with type 1 diabetes. Cardiovascular diabetology 2022;21(1):277; doi: 10.1186/s12933-022-01717-z.

19. Hoffman Robert P, Dye Amanda S, Huang Hong, et al. Effects of glucose control and variability on endothelial function and repair in adolescents with type 1 diabetes. ISRN endocrinology 2013;2013:876547; doi: 10.1155/2013/876547.

20. Kakuta Kentaro, Dohi Kaoru, Miyoshi Miho, et al. Impact of renal function on the underlying pathophysiology of coronary plaque composition in patients with type 2 diabetes mellitus. Cardiovascular diabetology 2017;16(1):131; doi: 10.1186/s12933-017-0618-3.

21. Koroleva EA, Korbut AI, Bulumbaeva DM, et al. Modeling of Risk Factors for Peripheral Artery Disease in Patients with Type 2 Diabetes. In: 2022 IEEE International Multi-Conference on Engineering, Computer and Information Sciences (SIBIRCON) IEEE: Yekaterinburg, Russian Federation; 2022; pp. 200–205; doi: 10.1109/sibircon56155.2022.10017018.

22. Lu Jingyi, Ma Xiaojing, Shen Yun, et al. Time in Range Is Associated with Carotid Intima-Media Thickness in Type 2 Diabetes. Diabetes technology & therapeutics 2020;22(2):72–78; doi: 10.1089/dia.2019.0251.

23. Magri Caroline Jane, Mintoff Dillon, Camilleri Liberato, et al. Relationship of Hyperglycaemia, Hypoglycaemia, and Glucose Variability to Atherosclerotic Disease in Type 2 Diabetes. Journal of diabetes research 2018;2018:7464320; doi: 10.1155/2018/7464320.

24. Mesa Alex, Gimenez Marga, Pueyo Irene, et al. Hyperglycemia and hypoglycemia exposure are differentially associated with micro- and macrovascular complications in adults with Type 1 Diabetes. Diabetes research and clinical practice 2022;189:109938; doi: 10.1016/j.diabres.2022.109938.

25. Mo Yifei, Zhou Jian, Li Mei, et al. Glycemic variability is associated with subclinical atherosclerosis in Chinese type 2 diabetic patients. Cardiovascular diabetology 2013;12:15; doi: 10.1186/1475-2840-12-15.

26. Morandi Anita, Piona Claudia, Corradi Massimiliano, et al. Risk factors for pre-clinical atherosclerosis in adolescents with type 1 diabetes. Diabetes research and clinical practice 2023;198:110618; doi: 10.1016/j.diabres.2023.110618.

27. Pena Alexia S, Couper Jennifer J, Harrington Jennifer, et al. Hypoglycemia, but not glucose variability, relates to vascular function in children with type 1 diabetes. Diabetes technology & therapeutics 2012;14(6):457–62; doi: 10.1089/dia.2011.0229.

28. Pertseva N, Moshenets K. Definition of the relationships between ambulatory blood pressure characteristics and blood glucose levels in type 2 diabetes patients with well-controlled arterial hypertension. 2023;30(2).

29. Piona Claudia, Marigliano Marco, Mancioppi Valentina, et al. Glycemic variability and Time in range are associated with the risk of overweight and high LDL-cholesterol in children and youths with Type 1 Diabetes. Hormone research in paediatrics 2023; doi: 10.1159/000535554.

30. Pulkkinen Mari-Anne, Tuomaala Anna-Kaisa, Hero Matti, et al. Motivational Interview to improve vascular health in Adolescents with poorly controlled type 1 Diabetes (MIAD): a randomized controlled trial. BMJ open diabetes research & care 2020;8(1); doi: 10.1136/bmjdrc-2020-001216.

31. Snell-Bergeon J K, Roman R, Rodbard D, et al. Glycaemic variability is associated with coronary artery calcium in men with Type 1 diabetes: the Coronary Artery Calcification in Type 1 Diabetes study. Diabetic medicine : a journal of the British Diabetic Association 2010;27(12):1436–42; doi: 10.1111/j.1464-5491.2010.03127.x.

32. Taya Naohiro, Katakami Naoto, Mita Tomoya, et al. Associations of continuous glucose monitoring-assessed glucose variability with intima-media thickness and ultrasonic tissue characteristics of the carotid arteries: a cross-sectional analysis in patients with type 2 diabetes. Cardiovascular diabetology 2021;20(1):95; doi: 10.1186/s12933-021-01288-5.

33. Torimoto K, Okada Y, Mita T, et al. Association of Glycaemia Risk Index With Indices of Atherosclerosis: A Cross‐Sectional Study. Journal of Diabetes 2025;17(3); doi: 10.1111/1753-0407.70065.

34. Wakasugi Satomi, Mita Tomoya, Katakami Naoto, et al. Associations between continuous glucose monitoring-derived metrics and arterial stiffness in Japanese patients with type 2 diabetes. Cardiovascular diabetology 2021;20(1):15; doi: 10.1186/s12933-020-01194-2.

35. Wei Yinghua, Liu Chunyan, Liu Yanyu, et al. The association between time in the glucose target range and abnormal ankle-brachial index: a cross-sectional analysis. Cardiovascular diabetology 2022;21(1):281; doi: 10.1186/s12933-022-01718-y.

36. Yano Yuichiro, Hayakawa Manabu, Kuroki Kazuo, et al. Nighttime blood pressure, nighttime glucose values, and target-organ damages in treated type 2 diabetes patients. Atherosclerosis 2013;227(1):135–9; doi: 10.1016/j.atherosclerosis.2012.12.006.

37. Yokota Shun, Tanaka Hidekazu, Mochizuki Yasuhide, et al. Association of glycemic variability with left ventricular diastolic function in type 2 diabetes mellitus. Cardiovascular diabetology 2019;18(1):166; doi: 10.1186/s12933-019-0971-5.

38. Zhang X-G, Zhang Y-Q, Zhao D-K, et al. Relationship between blood glucose fluctuation and macrovascular endothelial dysfunction in type 2 diabetic patients with coronary heart disease. European review for medical and pharmacological sciences 2014;18(23):3593–600.

39. Zhang Chuangbiao, Tang Meili, Lu Xiaohua, et al. Relationship of ankle-brachial index, vibration perception threshold, and current perception threshold to glycemic variability in type 2 diabetes. Medicine 2020;99(12):e19374; doi: 10.1097/MD.0000000000019374.

40. Zhou Hui, Wang Wei, Shen Qiuyue, et al. Time in range, assessed with continuous glucose monitoring, is associated with brachial-ankle pulse wave velocity in type 2 diabetes: A retrospective single-center analysis. Frontiers in endocrinology 2022;13:1014568; doi: 10.3389/fendo.2022.1014568.
