# Supplementary material for: Continuous Glucose Monitoring–Derived Metrics and Cardiovascular Risk Among People With Diabetes: Systematic Scoping Review
Source: JMIR Diabetes. 2026 May 6;11:e89374. doi: 10.2196/89374 (PMC13148326; doi:10.2196/89374)
Supplement: Multimedia Appendix 3 [file diabetes-v11-e89374-s003.docx]

## Continuous glucose monitoring

- ADRR = Average daily risk range
- AUC = Area under the curve
- BGRI = Blood glucose risk index
- CONGA = Continuous overall net glycemic action
- CV = Coefficient of variation
- GMI = Glucose management indicator
- GRI = Glucose risk index
- GV = Glycemic variability
- HBGI = High blood glucose index
- Hypo = Hypoglycemic events
- LAGE = Largest amplitude of glycemic excursions
- LBGI = Low blood glucose index
- MAGE = Mean amplitude of glycemic excursions
- MBG = Mean blood glucose
- MODD = Mean of daily differences
- PPGE = Postprandial glucose excursion
- SD = Standard deviation
- TAR = Time above range
- TBR = Time below range
- TIR = Time in range
- TITR = Time in tight range

## Clinical cardiovascular disease

- CAD = Coronary artery disease
  - CHD = Coronary heart disease
  - MI = Myocardial infarction
  - Heart attack
  - Heart failure
  - (Unstable) angina pectoris
- Cardiovascular events = Cardiovascular events or Ishemic heart disease
- CVD = Cardiovascular disease or Macrovascular complications or diabetic macroangiopathy
- LEAD = Lower extremity artery disease
- MACE = Major adverse cardiovascular events
- PAD = Peripheral artery disease
- Stroke = Stroke, Cerebrovascular disease, or Cerebrovascular accident

## Subclinical cardiovascular disease

- ABI = Ankle–brachial index
- BP = Blood pressure
- Carotid artery distensibility = Carotid artery distensibility or carotid distensibility coefficient
- CCA = Common carotid artery
- CIMT = Carotid artery intima-media thickness
- DBP = Diastolic blood pressure
- FMD = Flow-mediated dilation of the brachial artery
- GSM = Gray-scale median of the carotid arteries
- HRV = Heart rate variability
- IMT = Intima-media thickness
- PWV = Pulse wave velocity
  - ba-PWV = Brachial-ankle pulse wave velocity
  - cr-PWV = Carotid-radial pulse wave velocity
  - cf-PWV = Carotid-femoral pulse wave velocity
  - Aortic PWV = Aortic pulse wave velocity
- SBP = Systolic blood pressure
