# Supplementary material for: Continuous Glucose Monitoring–Derived Metrics and Cardiovascular Risk Among People With Diabetes: Systematic Scoping Review
Source: JMIR Diabetes. 2026 May 6;11:e89374. doi: 10.2196/89374 (PMC13148326; doi:10.2196/89374)
Supplement: Multimedia Appendix 5 [file diabetes-v11-e89374-s005.docx]

**Multimedia Appendix 5**

**Studies excluded during full-text screening**

## **Table S1**: Records from database searches:

| **Reference** | **Reason for exclusion** |
| --- | --- |
| Barbieri M, Rizzo MR, Marfella R, Boccardi V, Esposito A, Pansini A, Paolisso G. Decreased carotid atherosclerotic process by control of daily acute glucose fluctuations in diabetic patients treated by DPP-IV inhibitors. Atherosclerosis. 2013 Apr;227(2):349-54. | Not CGM metric |
| Cai J, Liu J, Lu J, Ni J, Wang C, Chen L, Lu W, Zhu W, Xia T, Zhou J. Impact of time in tight range on all-cause and cardiovascular mortality in type 2 diabetes: A prospective cohort study. Diabetes Obes Metab. 2025 Apr;27(4):2154-62. | Studies post-surgery |
| Catargi B, Gerbaud E. Glucose fluctuations and hypoglycemia as new cardiovascular risk factors in diabetes. Ann Endocrinol (Paris). 2021 Jun;82(3-4):144-5. | Not original article |
| Chernikova NA, Kamynina LL, Ametov AS. [The сardiometabolic assessment of the glycemic variability in patients with diabetes mellitus: the role of the glucocardiomonitoring]. Kardiologiia. 2020 Jun 3;60(5):902. | Not CVD outcome |
| Costantino S, Paneni F, Battista R, Castello L, Capretti G, Chiandotto S, Tanese L, Russo G, Pitocco D, Lanza GA, Volpe M, Lüscher TF, Cosentino F. Impact of Glycemic Variability on Chromatin Remodeling, Oxidative Stress, and Endothelial Dysfunction in Patients With Type 2 Diabetes and With Target HbA1c Levels. Diabetes. 2017 Sep;66(9):2472-82. | Not CVD outcome |
| Ehrmann D, Chatwin H, Schmitt A, Soeholm U, Kulzer B, Axelsen JL, Broadley M, Haak T, Pouwer F, Hermanns N. Reduced heart rate variability in people with type 1 diabetes and elevated diabetes distress: Results from the longitudinal observational DIA-LINK1 study. Diabet Med. 2023 Apr;40(4):e15040. | Not CVD outcome |
| Howsawi AA, Alem MM. Clinical implications and pharmacological considerations of glycemic variability in patients with type 2 diabetes mellitus. Sci Rep. 2024 Oct 14;14(1):24062. | Not CGM metric |
| Huang J, Zhang X, Li J, Tang L, Jiao X, Lv X. Impact of glucose fluctuation on acute cerebral infarction in type 2 diabetes. Can J Neurol Sci. 2014 Jul;41(4):486-92. | Studies post-surgery |
| Inaba Y, Tsutsumi C, Haseda F, Fujisawa R, Mitsui S, Sano H, Terasaki J, Hanafusa T, Imagawa A. Impact of glycemic variability on the levels of endothelial progenitor cells in patients with type 1 diabetes. Diabetol Int. 2017 Sep 4;9(2):113-20. | Not CVD outcome |
| Ito T, Ichihashi T, Fujita H, Sugiura T, Yamamoto J, Kitada S, Nakasuka K, Kawada Y, Ohte N. The impact of intraday glucose variability on coronary artery spasm in patients with dysglycemia. Heart Vessels. 2019 Aug;34(8):1250-7. | Studies post-surgery |
| Karnebeek K, Rijks JM, Dorenbos E, Gerver WM, Plat J, Vreugdenhil ACE. Changes in Free-Living Glycemic Profiles after 12 Months of Lifestyle Intervention in Children with Overweight and with Obesity. Nutrients. 2020 Apr 26;12(5):1228. | Not CVD outcome |
| Kietsiriroje N, Pearson SM, O'Mahoney LL, West DJ, Ariëns RA, Ajjan RA, Campbell MD. Glucose variability is associated with an adverse vascular profile but only in the presence of insulin resistance in individuals with type 1 diabetes: An observational study. Diab Vasc Dis Res. 2022 May-Jun;19(3):14791641221103217. | Not CVD outcome |
| Klimontov VV, Koroleva EA, Khapaev RS, Korbut AI, Lykov AP. Carotid Artery Disease in Subjects with Type 2 Diabetes: Risk Factors and Biomarkers. J Clin Med. 2021 Dec 24;11(1):72. | Not CGM metric |
| Koroleva EA, Khapaev RS, Lykov AP, Korbut AI, Klimontov VV. Association of carotid atherosclerosis and peripheral artery disease in patients with type 2 diabetes: risk factors and biomarkers. Diabetes Mellitus. 2023;26(2):172-81. Russian. | Language not understood |
| Maiorino MI, Della Volpe E, Olita L, Bellastella G, Giugliano D, Esposito K. Glucose variability inversely associates with endothelial progenitor cells in type 1 diabetes. Endocrine. 2015 Feb;48(1):342-5. | Not CVD outcome |
| Musurakis C, Poudel B, Chitrakar S, Qureshi F, Gilden JL. Evaluation Of Glucose Variability By Continuous Glucose Monitoring In Patients With Diabetes Mellitus And History Of Stroke. J Endocr Soc. 2023 Oct-Nov;7(Suppl 1): bvad114.970. | Not original article |
| Singh S, Singh DP, Singh SS, Srivastava AK. High Glycaemic Variability is a Pro-arrhythmic Factor in Patient of Type-2 Diabetes Mellitus With Heart Failure. Int J Diabetes Dev Ctries. 2019 Nov;39(Suppl 1):S1-S42. | Not original article |
| Stahn A, Pistrosch F, Ganz X, Teige M, Koehler C, Bornstein S, Hanefeld M. Relationship between hypoglycemic episodes and ventricular arrhythmias in patients with type 2 diabetes and cardiovascular diseases: silent hypoglycemias and silent arrhythmias. Diabetes Care. 2014 Feb;37(2):516-20. | Not CVD outcome |
| Sugimoto T, Saji N, Omura T, Tokuda H, Miura H, Kawashima S, Ando T, Nakamura A, Uchida K, Matsumoto N, Fujita K, Kuroda Y, Crane PK, Sakurai T. Cross-sectional association of continuous glucose monitoring-derived metrics with cerebral small vessel disease in older adults with type 2 diabetes. Diabetes Obes Metab. 2024 Aug;26(8):3318-27. | Not CVD outcome |
| Tang X, Li S, Wang Y, Wang M, Yin Q, Mu P, Lin S, Qian X, Ye X, Chen Y. Glycemic variability evaluated by continuous glucose monitoring system is associated with the 10-y cardiovascular risk of diabetic patients with well-controlled HbA1c. Clin Chim Acta. 2016 Oct 1;461:146-50. | Not CVD outcome |
| Tateishi K, Saito Y, Kitahara H, Kobayashi Y. Impact of glycemic variability on coronary and peripheral endothelial dysfunction in patients with coronary artery disease. J Cardiol. 2022 Jan;79(1):65-70. | Not CVD outcome |
| Tong L, Chi C, Zhang Z. Association of various glycemic variability indices and vascular outcomes in type-2 diabetes patients: A retrospective study. Medicine (Baltimore). 2018 May;97(21):e10860. | Not CGM metric |
| Wang X, Zhao X, Dorje T, Yan H, Qian J, Ge J. Glycemic variability predicts cardiovascular complications in acute myocardial infarction patients with type 2 diabetes mellitus. Int J Cardiol. 2014 Mar 15;172(2):498-500. | Studies post-surgery |
| Xia J, Xu J, Hu S, Hao H, Yin C, Xu D. Impact of glycemic variability on the occurrence of periprocedural myocardial infarction and major adverse cardiovascular events (MACE) after coronary intervention in patients with stable angina pectoris at 6months follow-up. Clin Chim Acta. 2017 Aug;471:196-200. | Not CGM metric |
| Yamamoto K, Ito T, Nagasato T, Shinnakasu A, Kurano M, Arimura A, Arimura H, Hashiguchi H, Deguchi T, Maruyama I, Nishio Y. Effects of glycemic control and hypoglycemia on Thrombus formation assessed using automated microchip flow chamber system: an exploratory observational study. Thromb J. 2019 Sep 2;17:17. | Not CVD outcome |
| Yang XJ, He H, Lü XF, Wen XR, Wang C, Chen DW, Li XJ, Ran XW. [Association of glycaemic variability and carotid intima-media thickness in patients with type 2 diabetes mellitus]. Sichuan Da Xue Xue Bao Yi Xue Ban. 2012 Sep;43(5):734-8. Chinese. | Language not understood |
| Yang X, Su G, Zhang T, Yang H, Tao H, Du X, Dong J. Comparison of admission glycemic variability and glycosylated hemoglobin in predicting major adverse cardiac events among type 2 diabetes patients with heart failure following acute ST-segment elevation myocardial infarction. J Transl Int Med. 2024 May 21;12(2):188-196. | Studies post-surgery |
| Yu MG, Jangolla SVT, Ziemniak N, Viebranz E, Chokshi T, Park K, Wu IH, Shah H, King GL. Cardiac Imaging And Metabolomics Studies Suggest Differential Risk And Protective Biomarkers For Cardiovascular Disease In Renoprotected, Long-Duration Type 1 Diabetes. J Endocr Soc. 2024 Oct-Nov;8(Suppl 1):bvae163.691. | Other reason (not yet published) |
| Zhang J, Li L, Zhai H, Chen H, Wang L, Li N, Liu R, Xia Y. Impact of blood glucose fluctuation on endothelial dysfunction and severity of stenosis in aged acute coronary syndrome patients. Int J Clin Exp Med. 2016;9(11):2210-9. | Studies post-surgery |
| Zhang J, Yang J, Liu L, Li L, Cui J, Wu S, Tang K. Significant abnormal glycemic variability increased the risk for arrhythmias in elderly type 2 diabetic patients. BMC Endocr Disord. 2021 Apr 27;21(1):83. | Not CVD outcome |

## **Table S2**: Records from citation search:

| **Reference** | **Reason for exclusion** |
| --- | --- |
| Gohbara M, Hibi K, Mitsuhashi T, Maejima N, Iwahashi N, Kataoka S, Akiyama E, Tsukahara K, Kosuge M, Ebina T, Umemura S, Kimura K. Glycemic Variability on Continuous Glucose Monitoring System Correlates With Non-Culprit Vessel Coronary Plaque Vulnerability in Patients With First-Episode Acute Coronary Syndrome - Optical Coherence Tomography Study. Circ J. 2016;80(1):202-10. | Studies post-surgery |
| Jin SM, Kim TH, Bae JC, Hur KY, Lee MS, Lee MK, Kim JH. Clinical factors associated with absolute and relative measures of glycemic variability determined by continuous glucose monitoring: an analysis of 480 subjects. Diabetes Res Clin Pract. 2014 May;104(2):266-72. | CGM metric as outcome |
| Kataoka S, Gohbara M, Iwahashi N, Sakamaki K, Nakachi T, Akiyama E, Maejima N, Tsukahara K, Hibi K, Kosuge M, Ebina T, Umemura S, Kimura K. Glycemic Variability on Continuous Glucose Monitoring System Predicts Rapid Progression of Non-Culprit Lesions in Patients With Acute Coronary Syndrome. Circ J. 2015;79(10):2246-54. | Studies post-surgery |
| Sugimoto H, Hironaka K, Yamada T, Otowa-Suematsu N, Hirota Y, Otake H, Hirata K, Sakaguchi K, Ogawa W, Kuroda S. Three components of glucose dynamics – value, variability, and autocorrelation – are independently associated with coronary plaque vulnerability. medRxiv 2023.11.21.23298816. | Other reason (preprint) |
