# Supplementary material for: Continuous Glucose Monitoring–Derived Metrics and Cardiovascular Risk Among People With Diabetes: Systematic Scoping Review
Source: JMIR Diabetes. 2026 May 6;11:e89374. doi: 10.2196/89374 (PMC13148326; doi:10.2196/89374)
Supplement: Multimedia Appendix 6 [file diabetes-v11-e89374-s006.docx]

**Multimedia Appendix 6**

To identify the geographical location of the studies, the hospitals and centers where data was collected were used. When it wasn’t possible to identify the geographical locations, the first author's affiliation was used as a proxy.

**Asia (n=27)**

- China (n=19)
- Japan (n=8)

**Europe (n=22)**

- Italy (n=7)
- 3 Belgium (n=3)
- 2 Ukraine (n=2)
- 2 Finland (n=2)
- 2 Spain (n=2)
- 1 Greece (n=1)
- 1 Malta (n=1)
- 1 Sweden (n=1)
- 1 The Netherlands (n=1)
- 1 Portugal (n=1)
- 1 Russia (n=1)

**North America (n=2)**

- 2 USA (n=2)

**Australia / Oceania (n=1)**

- 1 Australia (n=1)

**Borg 2011**(ADAG study) had study centers in:

- Europe: Netherlands, Denmark, Italy
- Africa: Cameroon
- North America: USA

Borg R, Kuenen J C, Carstensen B, Zheng H, Nathan D M, Heine R J, et al. HbA1(c) and

mean blood glucose show stronger associations with cardiovascular disease risk factors than do postprandial glycaemia or glucose variability in persons with diabetes: the A1C-Derived Average Glucose (ADAG) study. 2011; ADAG Study Group, editor. Diabetologia. 54: 69–72. doi:[10.1007/s00125-010-1918-2](https://doi.org/10.1007/s00125-010-1918-2)
