# Supplementary material for: Continuous Glucose Monitoring–Derived Metrics and Cardiovascular Risk Among People With Diabetes: Systematic Scoping Review
Source: JMIR Diabetes. 2026 May 6;11:e89374. doi: 10.2196/89374 (PMC13148326; doi:10.2196/89374)
Supplement: Multimedia Appendix 8 [file diabetes-v11-e89374-s008.docx]

**Multimedia Appendix 8**

List over how adjusted variables were grouped for the adjusted column in the results tables in the main manuscript

**Demographic**

- Age
- Sex
- Gender
- Age ≥ 65
- Education level

**Anthropometric**

Variables that describe body size or composition:

- Height z-score
- Weight (if it appears)
- BMI
- BMI z-score
- Body mass index (BMI)
- Waist circumference
- Body surface area

**Lifestyle**

- Smoking/ Smoking status/ Smoking pack years
- Alcohol use/ Alcohol history
- Physical activity (if it had appeared)

**Medical History**

- Diabetes duration
- Duration of pump therapy
- History of cancer
- Hypertension
- Dyslipidemia
- Diabetic retinopathy
- Use of ACEi or ARBs
- Past medical history
- Chronic heart failure
- Presence of retinopathy
- 3P MACE history
- Hypoglycemia unawareness
- Hyperlipidemia
- Atrial fibrillation (AF) type

**Physiological**

Vital signs or systemic functions:

- Blood pressure
- Systolic blood pressure
- Diastolic blood pressure
- SBP and DBP z-score
- Mean arterial pressure
- Heart rate
- Office systolic BP or brachial pulse pressure

**Biochemical**Blood/urine analytes, metabolic markers:

- HbA1c
- Fasting plasma glucose (FPG)
- Fasting insulin
- Triglycerides
- HDL-C
- LDL-C
- Total cholesterol
- Uric acid
- eGFR
- Urinary albumin-to-creatinine ratio
- Serum uric acid
- ALT, AST
- HOMA-IR, HOMA-β
- Log-transformed urine albumin
- ACR
- 5 Anhydroglucitol
- 2-h PG/insulin from OGTT
- hs-CRP
- Mean HbA1c over last 5 years
- Erythrocyte sedimentation rate

**Medication**

- Antiplatelet agents
- Metformin
- Use of antihypertensive drugs
- Use of statins
- Statin exposure
- Insulin therapy
- Insulin type
- anticoagulant use

**CGM/GV metrics**Variables related to CGM data:

- MAGE, TIR, TBR, MODD, SDBG, SD, CV
- AUC for BG < 3.9 mmol/L
- Lowest blood glucose
- M-value
- Number of days between CGM and CAC

**CVD marker**

- CHA2DS2-VASc score
- Index values for carotid atherosclerosis
- LVEF
- LVMI
