# Supplementary material for: Continuous Glucose Monitoring–Derived Metrics and Cardiovascular Risk Among People With Diabetes: Systematic Scoping Review
Source: JMIR Diabetes. 2026 May 6;11:e89374. doi: 10.2196/89374 (PMC13148326; doi:10.2196/89374)

# Multimedia Appendix 9

Table S1: Summary of reported associations between CGM-derived metrics and cardiovascular outcomes, categorized by statistical significance for all studies and stratified for T1D and T2D.

| **CGM-derived metrics:** | **TIR** | **MAGE** | **MBG** | **SD** | **CV** | **TBR** | **TAR** |
| --- | --- | --- | --- | --- | --- | --- | --- |
| **Total, N** | 23 | 22 | 21 | 19 | 19 | 16 | 15 |
| T1D, n | 10 | 5 | 9 | 7 | 8 | 9 | 8 |
| T2D, n | 11 | 14 | 8 | 10 | 8 | 7 | 7 |
| T1D, T2D and/or predabetes | 2 | 3 | 4 | 2 | 3 | 0 | 0 |
| **Association detected**  **(p<0.05), n(%)** | 7 (30%) | 14 (64%) | 9 (43%) | 5 (26%) | 4 (21%) | 2 (13%) | 2 (13%) |
| T1D, n(%) | 0 (0%) | 2 (40%) | 3 (33%) | 1 (14%) | 0 (0%) | 1 (11%) | 1 (13%) |
| T2D, n(%) | 5 (45%) | 10 (71%) | 4 (50%) | 4 (40%) | 2 (25%) | 1 (14%) | 1 (14%) |
| **Association not detected**  **(p>0.05), n(%)** | 8 (35%) | 6 (27%) | 11(52%) | 13(68%) | 12(63%) | 12 (75%) | 10(67%) |
| T1D, n(%) | 8 (80%) | 3 (60%) | 6 (67%) | 6 (86%) | 8 (100%) | 8 (89%) | 7 (88%) |
| T2D, n(%) | 0 (0%) | 2 (14%) | 3 (38%) | 5 (50%) | 3 (38%) | 4 (57%) | 3 (43%) |
| **Diverging findings n(%)** | 8 (35%) | 2 (9%) | 1 (5%) | 1 (5%) | 3 (16%) | 2 (13%) | 3 (20%) |
| T1D, n(%) | 3 (20%) | 0 (0%) | 0 (0%) | 0 (0%) | 0 (0%) | 0 (0%) | 0 (0%) |
| T2D, n(%) | 6 (55%) | 2 (14%) | 1 (13%) | 1 (10%) | 3 (38%) | 2 (29%) | 3 (43%) |
| **Total study size for the CGM-derived metrics, median [IQR]** | 445  [157-577] | 156  [63-344] | 105  [71-427] | 267  [77-480] | 342  [98-577] | 123  [77-312] | 152  [77-425] |
| T1D, median [IQR] | 214  [119-547] | 57  [30-215] | 105  [31-347] | 267  [54-515] | 152  [91-515] | 124  [102-161] | 138  [98-188] |
| T2D, median [IQR] | 510  [405-600] | 251  [89-411] | 405  [77-499] | 371  [95-484] | 499  [358-632] | 121  [75-499] | 405  [75-499] |

Abbreviations: MAGE, mean amplitude of glycemic excursions; TIR, time in range; MBG, mean blood glucose; SD, standard deviation; CV, coefficient of variation; TBR, time below range; TAR, time above range.

Table S2: Number of CGM metrics across all studies

| **Total**  **CGM metrics** | **N** | **Association detected (p<0.05),**  **n(%)** | **Association not detected (p>0.05),**  **n(%)** | **n, multiple outcomes & diverging findings** |
| --- | --- | --- | --- | --- |
| **TIR** | 23 | 7 (30%) | 8 (35%) | 8 (35%) |
| **MAGE** | 22 | 14 (64%) | 6 (27%) | 2 (9%) |
| **MBG** | 21 | 9 (43%) | 11 (52%) | 1 (5%) |
| **SD** | 19 | 5 (26%) | 13 (68%) | 1 (5%) |
| **CV** | 19 | 4 (21%) | 12 (63%) | 3 (16%) |
| **TBR** | 16 | 2 (13%) | 12 (75%) | 2 (13%) |
| **TAR** | 15 | 2 (13%) | 10 (67%) | 3 (30%) |

Table S3: Number of CGM metrics across studies only including T1D

| **T1D**  **CGM metrics** | **N** | **Association detected (p<0.05),**  **n(%)** | **Association not detected (p>0.05),**  **n(%)** | **n, multiple outcomes & diverging findings** |
| --- | --- | --- | --- | --- |
| **TIR** | 11 | 0 (0%) | 8 (80%) | 2 (20%) |
| **MAGE** | 5 | 2 (40%) | 3 (60%) | 0 (0%) |
| **MBG** | 9 | 3 (33%) | 6 (67%) | 0 (0%) |
| **SD** | 7 | 1 (14%) | 6 (86%) | 0 (0%) |
| **CV** | 8 | 0 (0%) | 8 (100%) | 0 (0%) |
| **TBR** | 9 | 1 (11%) | 8 (89%) | 0 (0%) |
| **TAR** | 8 | 1 (13%) | 7 (88%) | 0 (0%) |

Table S4: Number of CGM metrics across studies only including T2D

| **T2D**  **CGM metrics** | **N** | **Association detected (p<0.05),**  **n(%)** | **Association not detected (p>0.05),**  **n(%)** | **n, multiple outcomes & diverging findings** |
| --- | --- | --- | --- | --- |
| **TIR** | 11 | 5 (45%) | 0 (9%) | 6 (55%) |
| **MAGE** | 14 | 10 (71%) | 2 (14%) | 2 (14%) |
| **MBG** | 8 | 4 (50%) | 3 (38%) | 1 (13%) |
| **SD** | 10 | 4 (40%) | 5 (50%) | 1 (10%) |
| **CV** | 8 | 2 (25%) | 3 (38%) | 3 (38%) |
| **TBR** | 7 | 1 (14%) | 4 (57%) | 2 (29%) |
| **TAR** | 7 | 1 (14%) | 3 (43%) | 3 (43%) |

Table S5: Number of CGM metrics across studies including both T1D and T2D, or T2D and prediabetes, or T1D, T2D and prediabetes

| **T1D, T2D and prediabetes.**  **CGM metrics** | **N** | **Association detected (p<0.05),**  **n(%)** | **Association not detected (p>0.05),**  **n(%)** | **n, multiple outcomes & diverging findings** |
| --- | --- | --- | --- | --- |
| **TIR** | 2 | 2 | 0 | 0 |
| **MAGE** | 3 | 2 | 1 | 0 |
| **MBG** | 4 | 2 | 2 | 0 |
| **SD** | 2 | 0 | 2 | 0 |
| **CV** | 3 | 2 | 1 | 0 |
| **TBR** | 0 | 0 | 0 | 0 |
| **TAR** | 0 | 0 | 0 | 0 |

# Calculations of study size populations


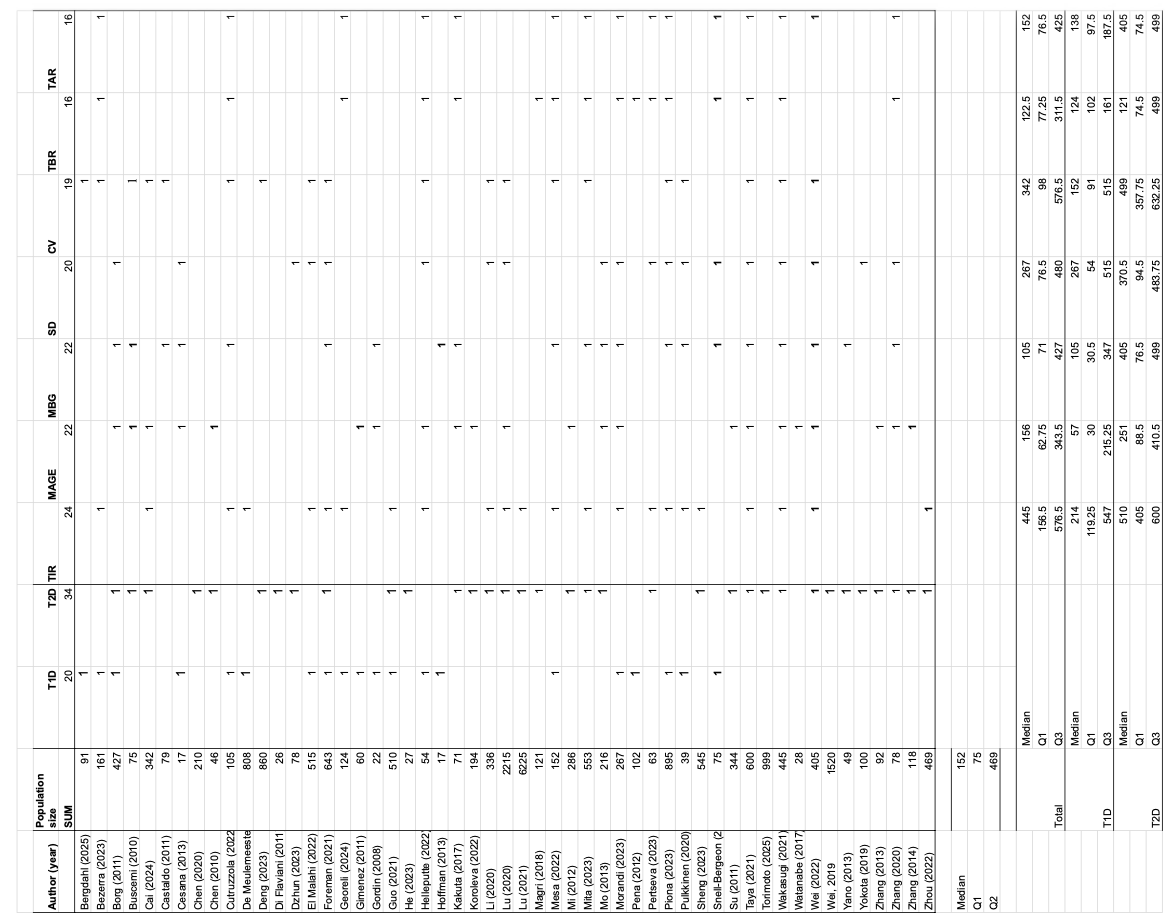

Supplement: Multimedia Appendix 9 [file diabetes-v11-e89374-s009.docx]
