# Supplementary material for: Continuous Glucose Monitoring–Derived Metrics and Cardiovascular Risk Among People With Diabetes: Systematic Scoping Review
Source: JMIR Diabetes. 2026 May 6;11:e89374. doi: 10.2196/89374 (PMC13148326; doi:10.2196/89374)
Supplement: Multimedia Appendix 10 [file diabetes-v11-e89374-s010.docx]

**Multimedia Appendix 10.** Full table (with adjustments) of the main findings of the included studies on clinical cardiovascular outcomes. The details of the references are available in Multimedia Appendix 4.

| **Outcome** | **Author, year** | **CGM metrics** | **Unadjusted or least adjusted** | **p-value for**  **lesst adjusted** | **Most adjusted** | **p - value for most adjusted** | **Adjustments** |
| --- | --- | --- | --- | --- | --- | --- | --- |
| Cardiovascular mortality | Lu, 2021 [21] | TIR >85%: | HR: 1.00 | p trend: <0.001 | HR: 1.00 | p trend: 0.015 | Model 1:  Demographic  Model 2:  Demographic  Anthropometric  Lifestyle  Medical History  Physiological  Biochemical  Medication |
|  |  | TIR: 71–85% | HR: 1.43 (0.95–2.14) |  | HR: 1.35 (0.90, 2.04) |  |  |
|  |  | TIR: 51–70% | HR: 1.66 (1.12–2.45) |  | HR: 1.47 (0.99, 2.19) |  |  |
|  |  | TIR ≥50% | HR: 2.15 (1.47–3.13) |  | HR: 1.85 (1.25, 2.72) |  |  |
|  |  | TIR as a continuous variable each 10% decrease | HR: 1.08 (1.03–1.13) | - | HR: 1.05 (1.00–1.11) | - |  |
|  | Wei, 2019* [46] | Hypoglycemic events | HR: 2.033 (1.211, 3.413) | nr | HR: 2.642 (1.398, 4.994) | nr | Least adjusted:  Unadjusted  Most adjusted:  Demographic Anthropometric Lifestyle Medical History Biochemical Medication |
| Major adverse cardiovascular events | He, 2023 [45] | BGRI | HR: 0.97 (0.85, 1.10) | 0.607 | HR: 0.98 (0.85, 1.13) | 0.746 | Least adjusted:  Unadjusted  Most adjusted:  Demographic  Anthropometric  Medical History |
|  |  | LBGI | HR: 2.37 (1.16, 4.83) | 0.018 | HR: 2.73 (1.21, 6.16) | 0.016 |  |
|  |  | HBGI | HR: 0.94 (0.81, 1.08) | 0.381 | HR: 0.94 (0.81, 1.09) | 0.436 |  |
|  |  | ADRR | HR: 1.00 (0.93, 1.07) | 0.995 | HR: 1.01 (0.93, 1.09) | 0.801 |  |
|  |  | GMI | HR: 0.98 (0.91, 1.06) | 0.651 | HR: 0.99 (0.91, 1.07) | 0.775 |  |
|  |  | M-value | HR: 0.98 (0.91, 1.05) | 0.535 | HR: 0.98 (0.91, 1.06) | 0.644 |  |
|  | Wei, 2019* [46] | Hypoglycemic events | HR: 1.501 (1.207, 1.866) | nr | HR: 1.615 (1.239, 2.106) | <0.001 | Least adjusted:  Unadjusted  Most adjusted:  Demographic Anthropometric Lifestyle Medical History Biochemical Medication |
| Macrovascular complications | De Meulemeester, 2024†* [48] | TIR | OR: 0.939 (0.829, 1.063) | p > 0.05 | OR: 0.896 (0.738, 1.087) | p > 0.05 | Least adjusted:  Unadjusted  Most adjusted:  Demographic Anthropometric Lifestyle Medical History Physiological Biochemical Medication |
|  |  | TITR | OR: 0.901 (0.775, 1.047) | p > 0.05 | OR: 0.933 (0.745, 1.169) | p > 0.05 |  |
|  | Bezerra, 2023 [47] | TIR | OR: 0.66 (0.46–0.93) | 0.019 | OR: 0.68 (0.39, 1.16), | 0.152 | Least adjusted:  Unadjusted  Most adjusted:  Demographic Medical History |
|  |  | TB54 | OR: 1.10 (0.88–1.38) | 0.388 | OR: 0.92 (0.62, 1.34) | 0.652 |  |
|  |  | TBR | OR: 0.93 (0.80–1.09) | 0.393 | OR: 0.77 (0.54, 1.11) | 0.166 |  |
|  |  | TAR | OR: 1.04 (1.01–1.08) | 0.010 | OR: 1.04 (0.99, 1.10) | 0.083 |  |
|  |  | TA250 | OR: 1.04 (1.00-1.08) | 0.029 | OR: 1.03 (0.97, 1.09) | 0.286 |  |
|  |  | CV | OR: 1.03 (0.95–1.11) | 0.524 | OR: 0.92 (0.81, 1.06) | 0.252 |  |
|  |  | GMI | OR: 2.17 (1.14–4.11) | 0.018 | OR: 2.03 (0.77, 5.37) | 0.154 |  |
|  | Deng, 2023 [49] | %CV Tertile 1 | Hp 1, OR: 1.000 (Reference) | p-value for interaction:  0.071 | Hp 1, OR: 1.000 (Reference) | p-value for interaction: 0.008 | Least adjusted:  Unadjusted  Most adjusted:  Demographic  Anthropometric  Medical History  Physiological  Biochemical |
|  |  |  | Hp 2-2, OR: 1.000 (Reference) |  | Hp 2-2, OR: 1.000 (Reference) |  |  |
|  |  | %CV Tertile 2 | Hp 1, OR: 483 (0.907, 2.423) | 0.116 | Hp 1, OR: 1.048 (0.528, 2.078) | 0.894 |  |
|  |  |  | Hp 2-2 , OR: 1.399 (0.829, 2.358) | 0.208 | Hp 2-2 , OR: 0.659 (0.296, 1.466) | 0.306 |  |
|  |  | %CV Tertile 3 | Hp 1, OR: 2.347 (1.393, 3.957) | 0.001 | Hp 1, OR: 2.461 (1.183, 5.121) | 0.016 |  |
|  |  |  | Hp 2-2, OR: 1.217 (0.731, 2.027) | 0.451 | Hp 2-2, OR: 0.540 (0.245, 1.191) | 0.127 |  |
|  | El Malahi, 2022 [50] | TIR | nr | nr | nr | p > 0.05 | Demographic Medical History CGM/GV metrics |
|  |  | SD |  |  | nr | p > 0.05 |  |
|  |  | CV |  |  | nr | p > 0.05 |  |
|  | Magri, 2018† [27] | TBR | nr | nr | OR: 1.12 (1.014–1.228) | 0.024 | Anthropometric Lifestyle Medical History Biochemical CGM/GV metrics |
|  |  | Lowest BG value |  |  | nr | ns |  |
|  |  | AUC under TBR |  |  | nr | ns |  |
| Coronary Artery Disease | De Meulemeester, 2024†* [48] | TITR | OR: 1.039 (0.812, 1.330) | p > 0.05 | OR: 1.255 (0.874, 1.803) | p > 0.05 | Least adjusted:  Unadjusted  Most adjusted:  Demographic Anthropometric Lifestyle Medical History Physiological Biochemical Medication |
|  |  | TIR | OR: 1.072 (0.866, 1.328) | p > 0.05 | OR: 1.164 (0.844, 1.607) | p > 0.05 |  |
|  | Sheng, 2023 [53] | TIR(%): <20 | nr | nr | OR: 2.143 (1.554–3.287) | nr | Study states that they adjust for confounders (The confounders are not reported) |
|  |  | TIR(%): 20–40 |  |  | OR: 1.049 (0.945–2.022) | nr |  |
|  |  | TIR(%): 40–60 |  |  | OR: 0.854 (0.495–1.473) | nr |  |
|  |  | TIR(%): 60–80 |  |  | OR: 0.617 (0.423–1.312) | nr |  |
|  |  | TIR(%): >80 |  |  | OR: 0.470 (0.143–1.545) | nr |  |
|  | Chen, 2020†* [40] | Controls with: SD <1.40 mmol/L MAGE <3.90 mmol/L LAGE <4.40 mmol/L MODD <0.83 mmol/L vs high BG fluctuations | N/A | N/A | Myocardial Infarction, 𝜒^2: 5.797 | 0.016 | N/A |
|  |  |  |  |  | Angina pectoris, 𝜒^2: 7.49 | 0.006 | N/A |
|  | Wei, 2019* [46] | Hypoglycemic events | MI, HR: 1.901 (1.067, 3.389) | nr | MI, HR: 1.549 (0.768, 3.124) | 0.030 | Least adjusted:  Unadjusted  Most adjusted:  Demographic Anthropometric Lifestyle Medical History Biochemical Medication |
|  |  | Hypoglycemic events | Unstable angina pectoris,  HR: 1.226 (0.857, 1.753) | nr | Unstable angina pectoris, HR: 1.218 (0.794, 1.869) | 0.300 |  |
|  | Mi, 2012†* [43] | MAGE level (≥3.4 mmol/L): | nr | nr | OR: 2.286(1.176, 4.446), | 0.015 | Demographic Lifestyle Medical History Biochemical CGM/GV metrics |
|  | Su, 2011* [28] | MAGE≥3.4 mmol/L | nr | nr | OR: 2.612 (1.423, 4.831) | 0.002 | Demographic  Lifestyle  Medical History  Biochemical  Medication |
|  |  | MAGE |  |  | AUC: 0.618 (0.555, 0.680) | 0.001 | N/A |
| Gensini score | Chen, 2020†* [40] | Controls with: SD <1.40 mmol/L MAGE <3.90 mmol/L LAGE <4.40 mmol/L MODD <0.83 mmol/L vs high BG fluctuations | N/A | N/A | 𝘵: 6.210 | p < 0.001 | N/A |
|  | Watanabe, 2017* [54] | MAGE |  |  | r = 0.742 | p < 0.001 | N/A |
|  | Mi, 2012†* [43] | MAGE | nr | nr | Unstandardized coefficients beta: 4.817, SE: 1.614 Standardized coefficients beta: 0.170, t = 2.984 | 0.003 | Demographic  Anthropometric  Physiological  Biochemical |
|  | Su, 2011* [28] | MAGE | nr | nr | Unstandardized beta: 7.010, SE: 1.466 Standardized beta: 0.237, t = 4.783 | <0.001 | Demographic Medical History Physiological Biochemical CGM/GV metrics |
| Syntax score | Watanabe, 2017* [54] | MAGE | N/A | N/A | r = 0.776 | p < 0.001 | N/A |
|  | Zhang, 2013* [30] | MAGE | N/A | N/A | r = 0.518 | 0.011 | N/A |
|  |  | BG fluctuations at 00:00 - 03:00 | N/A | N/A | r = −0.442 | 0.035 | N/A |
|  |  | BG fluctuations at 03:00 - 06:00 |  |  | r = −0.208 | 0.340 | N/A |
|  |  | BG fluctuations at 06:00 - 08:00 |  |  | r = 0.678 | <0.001 | N/A |
|  |  | BG fluctuations at 08:00 - 11:00 |  |  | r = 0.115 | 0.600 | N/A |
|  |  | BG fluctuations at 11:00 - 13:00 |  |  | r = 0.523 | 0.011 | N/A |
|  |  | BG fluctuations at 13:00 - 17:00 |  |  | r = 0.257 | 0.237 | N/A |
|  |  | BG fluctuations at 17:00 - 19:00 |  |  | r = 0.358 | 0.094 | N/A |
|  |  | BG fluctuations at 19:00 - 24:00 |  |  | r = −0.018 | 0.933 | N/A |
| Stroke | De Meulemeester, 2024†* [48] | TITR | OR: 0.651 (0.470, 0.902) | p < 0.05 | OR: 0.546 (0.347, 0.858) | p < 0.01 | Least adjusted:  Unadjusted  Most adjusted:  Demographic Anthropometric Lifestyle Medical History Physiological Biochemical Medication |
|  |  | TIR | OR: 0.749 (0.588, 0.955) | p < 0.05 | OR: 0.617 (0.440, 0.866) | p < 0.01 |  |
|  | Guo, 2021 [51] | TIR: Q1 (≤46%) | OR: 1.00 (Reference) | p < 0.001 | OR: 1.00 (Reference) | p < 0.001 | Least adjusted:  Univariate  Most adjusted:  Demographic  Anthropometric  Medical History  Biochemical  Medication  CVD marker |
|  |  | TIR: Q2 (46-65%) | 0.86 (0.72, 0.95) |  | OR: 0.80 (0.68, 0.92) |  |  |
|  |  | TIR: Q3 (65-81%) | 0.71 (0.61, 0.81) |  | OR: 0.64 (0.53, 0.79) |  |  |
|  |  | TIR: Q4 (>81%) | 0.66 (0.58, 0.80) |  | OR: 0.59 (0.50, 0.74) |  |  |
|  |  | TIR, per 10% increase | 0.93 (0.85, 0.98) 0.008 | 0.008 | OR: 0.89 (0.82, 0.95) | p = 0.001 | Model 1:  Demographic  Anthropometric  Model 3: Demographic Anthropometric Medical History Biochemical Medication CVD marker |
|  | Wei, 2019* [46] | Hypoglycemic events | HR: 1.691 (1.144, 2.499) | nr | HR: 1.813 (1.110, 2.960) | 0.060 | Least adjusted:  Unadjusted  Most adjusted:  Demographic Anthropometric Lifestyle Medical History Biochemical Medication |
| Peripheral Artery Disease | De Meulemeester, 2024†* [48] | TITR | OR: 0.680 (0.426, 1.085) | p > 0.05 | OR: 0.807 (0.382, 1.703) | p > 0.05 | Least adjusted:  Unadjusted  Most adjusted:  Demographic Anthropometric Lifestyle Medical History Physiological Biochemical Medication |
|  |  | TIR | OR: 0.736 (0.520, 1.042) | p > 0.05 | OR: 0.811 (0.470, 1.398) | p > 0.05 |  |
| Lower extremity arterial disease | Li, 2020 [52] | TIR | OR: 0.979 (0.968,0.991) | p < 0.001 | OR: 0.979 (0.965,0.992) | 0.002 | Least adjusted:  Unadjusted  Model 3: Demographic Anthropometric Lifestyle Medical History Physiological Biochemical Medication |
|  |  | CV | OR: 1.040 (1.003,1.078) | 0.035 | OR: 1.038 (0.996,1.081) | 0.075 |  |
|  |  | SD | OR: 1.325 (1.038,1.691) | 0.024 | OR: 1.158 (0.824,1.627) | 0.399 |  |
|  |  | TIR -> without LEAD | OR: 1.00 | Reference | OR: 1.00 | Reference | Model 1:  Demographic Anthropometric  Medical History Physiological  Biochemical  Model2: Demographic Anthropometric Medical History Physiological Biochemical SD metric |
|  |  | TIR -> mild LEAD | OR: 0.98 (0.97,1.00) | 0.142 | OR: 0.99 (0.97,1.01) | 0.250 |  |
|  |  | TIR -> moderate LEAD | OR: 0.97 (0.95,0.99) | 0.007 | OR: 0.97 (0.95,0.99) | 0.013 |  |
|  |  | TIR -> without severe | OR: 0.96 (0.94,0.98) | 0.002 | OR: 0.96 (0.94,0.98) | 0.003 |  |
|  |  | CV -> without LEAD | N/A | N/A | OR: 1.00 | Reference |  |
|  |  | CV -> mild LEAD |  |  | OR: 1.03 (0.98,1.07) | 0.280 |  |
|  |  | CV -> moderate LEAD |  |  | OR: 1.02 (0.96,1.09) | 0.478 |  |
|  |  | CV -> without severe |  |  | OR: 1.02 (0.95,1.09) | 0.598 |  |
|  |  | TIR -> without LEAD | N/A | N/A | OR: 1.00 | Reference | Model 3: Demographic Anthropometric Medical History Physiological Biochemical CV metric |
|  |  | TIR -> mild LEAD |  |  | OR: 0.97 (0.96,1.08) | 0.060 |  |
|  |  | TIR -> moderate LEAD |  |  | OR: 0.98 (0.95,0.99) | 0.013 |  |
|  |  | TIR -> without severe |  |  | OR: 0.97 (0.95,0.99) | 0.024 |  |
|  |  | SD -> without LEAD | N/A | N/A | OR: 1.00 | Reference |  |
|  |  | SD -> mild LEAD |  |  | OR: 0.88 (0.47,1.64) | 0.690 |  |
|  |  | SD -> moderate LEAD |  |  | OR: 1.28 (0.58,3.07) | 0.582 |  |
|  |  | SD -> without severe |  |  | OR: 1.52 (0.92,2.41) | 0.100 |  |

^* This study appears multiple times in the clinical cardiovascular disease outcome tables due to investigating multiple CVD outcomes.^

^† This study appears both in the clinical and subclinical disease outcome tables due to investigating multiple CVD outcomes^

^All hazard ratios (HR), odds ratios(OR) and AUCs are reported as: point estimate (95%CI)^

^Further elaboration on the adjusted variables can be found in Multimedia Appendices 7 and 8.^

^Abbreviations: CGM, continuous glucose monitoring; GV, glycemic variability; CV, coefficient of variation; SD, standard deviation; TIR, time in range; TBR, time below range; TB54, Time below 54 mg/dL; TAR, time above range; TA250, time above 250 mg/dL; MAGE, mean amplitude of glycemic excursions; GMI, glucose management indicator; LBGI, low blood glucose index; HBGI, high blood glucose index; ADRR, average of daily risk range; BGRI, Blood glucose risk index; TITR, time in tight range; MODD, mean of daily differences; LAGE, largest amplitude of glycemic excursions; PPGE, postprandial glucose excursions; CVD, cardiovascular disease; ct, cannot tell; N/A, not applicable; nr, not reported.^
